# Selective processing of all rotational and translational optic flow directions in the zebrafish pretectum and tectum

**DOI:** 10.1186/s12915-019-0648-2

**Published:** 2019-03-29

**Authors:** Kun Wang, Julian Hinz, Väinö Haikala, Dierk F. Reiff, Aristides B. Arrenberg

**Affiliations:** 1 Werner Reichardt Centre for Integrative Neuroscience, Institute of Neurobiology, University of Tübingen, 72076 Tübingen, Germany; 2 Graduate Training Centre for Neuroscience, University of Tübingen, 72076 Tübingen, Germany; 3 Present address: Friedrich Miescher Institute for Biomedical Research, 4058 Basel, Switzerland; 4 Neurobiology and Behavior, Institute Biology 1, Faculty of Biology, University of Freiburg, 79104 Freiburg, Germany

**Keywords:** Optomotor response, Optokinetic response, Pretectum, Optic tectum, Zebrafish, Optic flow, Binocular integration, Calcium imaging

## Abstract

**Background:**

The processing of optic flow in the pretectum/accessory optic system allows animals to stabilize retinal images by executing compensatory optokinetic and optomotor behavior. The success of this behavior depends on the integration of information from both eyes to unequivocally identify all possible translational or rotational directions of motion. However, it is still unknown whether the precise direction of ego-motion is already identified in the zebrafish pretectum or later in downstream premotor areas.

**Results:**

Here, we show that the zebrafish pretectum and tectum each contain four populations of motion-sensitive direction-selective (DS) neurons, with each population encoding a different preferred direction upon monocular stimulation. In contrast, binocular stimulation revealed the existence of pretectal and tectal neurons that are specifically tuned to only one of the many possible combinations of monocular motion, suggesting that further downstream sensory processing might not be needed to instruct appropriate optokinetic and optomotor behavior.

**Conclusion:**

Our results suggest that local, task-specific pretectal circuits process DS retinal inputs and carry out the binocular sensory computations necessary for optokinetic and optomotor behavior.

**Electronic supplementary material:**

The online version of this article (10.1186/s12915-019-0648-2) contains supplementary material, which is available to authorized users.

## Background

Animals process optic flow and execute optokinetic (OKR) and/or optomotor responses (OMR) to actively stabilize their gaze and position relative to the visual world [1–6]. The underlying sensorimotor transformations involve direction-selective retinal ganglion cells (DS-RGCs) that project to the pretectum/accessory optic system (AOS). The pretectum in turn projects to (pre-) motor midbrain and hindbrain areas to evoke compensatory eye and tail movements [1, 7–10]. The optic tectum also receives DS-RGC inputs and is involved in detecting the location of small visual stimuli, as needed during prey capture behavior [11–13]. The directional tuning of neurons in the zebrafish pretectum is still unknown, while the optic tectum has been shown to be represented by four preferred directions, roughly corresponding to up, down, temporal-to-nasal (TN), and nasal-to-temporal (NT) [12, 14, 15]. For the detection of ego-motion directions, comparing the motion information across the two eyes is an efficient strategy for lateral-eyed animals, which has been shown to be implemented in the zebrafish pretectum for certain directions [10]. Corresponding binocular neurons (selective for translational or rotational motion) have also been identified in other species [16, 17]. However, a recent study suggested that the hindbrain is necessary for the unambiguous identification of optic flow directions in zebrafish and reported the binocular sensory representation in the pretectum to be incomplete for this task [9].

Here, we investigated the binocular sensory representation in the main visual brain areas of zebrafish, the optic tectum and the pretectum. First, we characterized the direction selectivity of zebrafish pretectal and tectal neurons using monocular visual stimuli. Based on the preferred directions of these neurons, binocular stimulation was used to identify neurons selective for specific binocular visual stimulus combinations. For each of the six spatial degrees of freedom, we identified corresponding neurons selectively representing it. These binocular-selective neurons may evoke appropriate compensatory swim and eye movements by direct axonal projections to motor nuclei in the tegmentum and hindbrain [1, 18, 19]. Our results suggest that optic flow directions are readily detected in local, retinorecipient sensory circuits to support reflexive optomotor and optokinetic responses.

## Results

### Neurons of the pretectum and tectum prefer one of four cardinal motion directions during monocular stimulus presentation

**Fig. 1:**
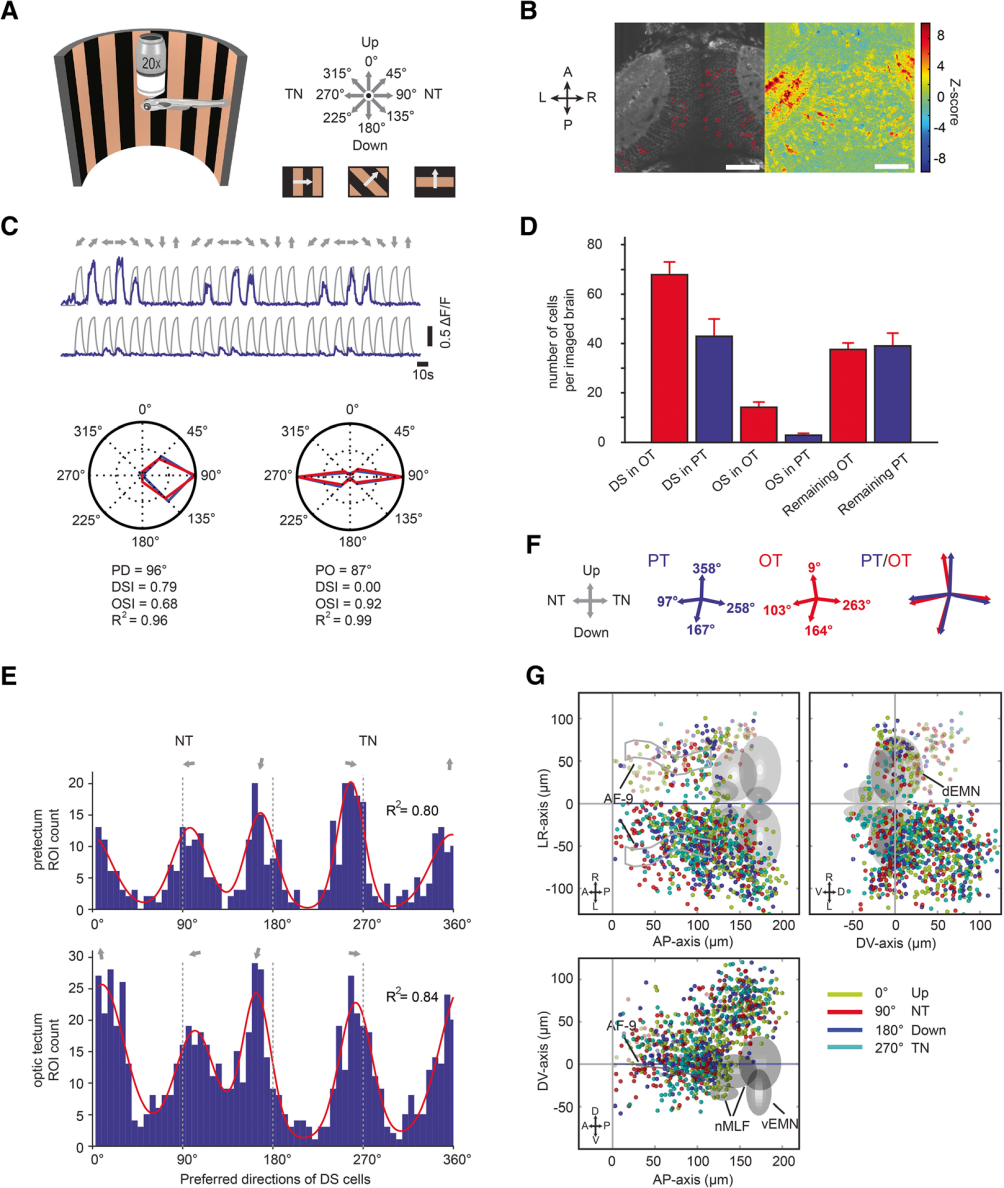
Monocular motion stimuli reveal four orthogonal preferred directions in the zebrafish pretectum and optic tectum. **a** A half-cylindrical stimulus arena was used to present motion in eight different directions to the zebrafish (not drawn to scale). **b** Time-averaged optical slice of tectal GCaMP5G expression (left). The z-score heat map (right) was used to detect motion-sensitive pixels and circular regions of interest (ROIs) were drawn manually (left). Scale bar, 50 μm (**c**) Top: ΔF/F responses (of three stimulus repetitions) of two example neurons. Grey lines indicate the motion phases; their shape corresponds to the signal expected for a motion-sensitive cell (regressor, see “Materials and methods”). Bottom: Polar plots illustrating responses for each stimulus phase for one direction-selective (left) and one orientation-selective (right) cell. Blue lines correspond to the median ΔF/F from three repetitions, red lines to the fitted von-Mises function used to infer the preferred direction (PD), the direction and orientation selectivity indices (DSI and OSI), and the goodness of fit (R^2^). **d** Number of identified DS and OS cells per recorded brain in optic tectum (OT) and pretectum (PT). Motion-sensitive cells that are neither DS nor OS are classified as “Remaining.” **e** Histograms of the preferred directions of direction-selective neurons in pretectum (top) and optic tectum (bottom) (pooled from nine imaged brains). The four peaks were fitted with a sum of four von-Mises functions (red line). **f** The four fitted peak directions from **e** are plotted for pretectum (blue) and optic tectum (red). Please note that in panels **e** and **f** the illustration arrow for NT points in a different direction than in panels **a** and **c**. We chose to switch arrangement to allow an easier comparison of panel (**e**) to the plots published in a previous report (Fig. 2a of Hunter et al [14]). **g** Anatomical maps of DS neurons (color-coded according to PD) in the tectum and pretectum. AF-9, arborization field 9-containing neuropil; nMLF, nucleus of the medial longitudinal fasciculus; vEMN/dEMN, ventral and dorsal extraocular motoneurons. Error bars correspond to SEM. A, anterior; P, posterior; L, left; R, right; D, dorsal; V, ventral

Since monocular motion processing likely forms the basis for the comparison of binocular motion patterns, we first set out to investigate the representation of preferred motion direction (PD) using monocular gratings moving in eight different directions. Grating motion was presented to the right eye of larval zebrafish (*HuC:GCaMP5G*) [20] that broadly expressed GCaMP5G in the brain. Stimulus-associated changes in the concentration of somatic calcium ions in neurons of the diencephalon and midbrain were recorded using two-photon population calcium imaging (Fig. 1a–c) [10]. Tectal and pretectal brain regions were identified based on anatomical landmarks (Additional file 1: Figure S1A). Identified motion-sensitive neurons were further classified based on their orientation- (OS) or direction-selective (DS) responses (calculation of orientation and direction selectivity indices, OSI and DSI). Neurons were considered DS if the DSI was larger than 0.7 (Additional file 1: Figure S1C), and OS if OSI > 0.5 and DSI < 0.7. About half of the motion-sensitive neurons were DS (pretectum, 50 ± 4% (43/85); tectum, 56 ± 3% (68/120); mean ± SEM across fish, *n* = 9 for both pretectum and tectum), and a minority were OS (pretectum, 3 ± 0.8% (3/85); tectum, 12 ± 1.8% (14/120); *n* = 9 fish). We identified 43 ± 7 DS neurons per recorded pretectum and 68 ± 5 DS cells per recorded tectum (Fig. 1d). The tuning specificity of DS neurons, measured by the normalized vector sum, varied greatly across neurons (Additional file 1: Figure S1D).

Tectal DS neurons were each tuned to one out of four preferred directions of motion (Fig. 1e), corresponding to up (9°), down (164°), TN (263°), and NT (103°). The observed preferred directions are comparable to those reported in a previous study [14]. We found that the four classes of DS neurons were approximately equally abundant (Fig. 1e), while horizontal directions were more prominently represented in the previous study [14]. Notably, DS neurons of the pretectum preferred the same directions (up, 358°; down, 167°; TN, 258°; NT, 97°). Intriguingly, the preferred directions of neurons in both the pretectum and tectum were not precisely orthogonal to each other: the angle between down and NT was smaller (~ 70°) and the angle between up and TN was larger (~ 100°) than 90° (Fig. 1e, f).

Tectal and pretectal DS neurons were predominantly identified in the brain hemisphere contralateral to the stimulated eye (laterality indices for tectum, 0.88 ± 0.04; pretectum, 0.70 ± 0.12; with a value of − 1 corresponding to an exclusively ipsilateral and + 1 to an exclusively contralateral placement of neurons). No salient anatomical clustering of functionally similar DS neurons was observed (Fig. 1g). In summary, our monocular stimulation experiments showed that the four Cartesian axes of motion are well represented in both tectum and pretectum.

### Pretectal and tectal neurons encode specific binocular optic flow patterns corresponding to rotation or translation about or along the yaw, roll, or pitch axes

**Fig. 2:**
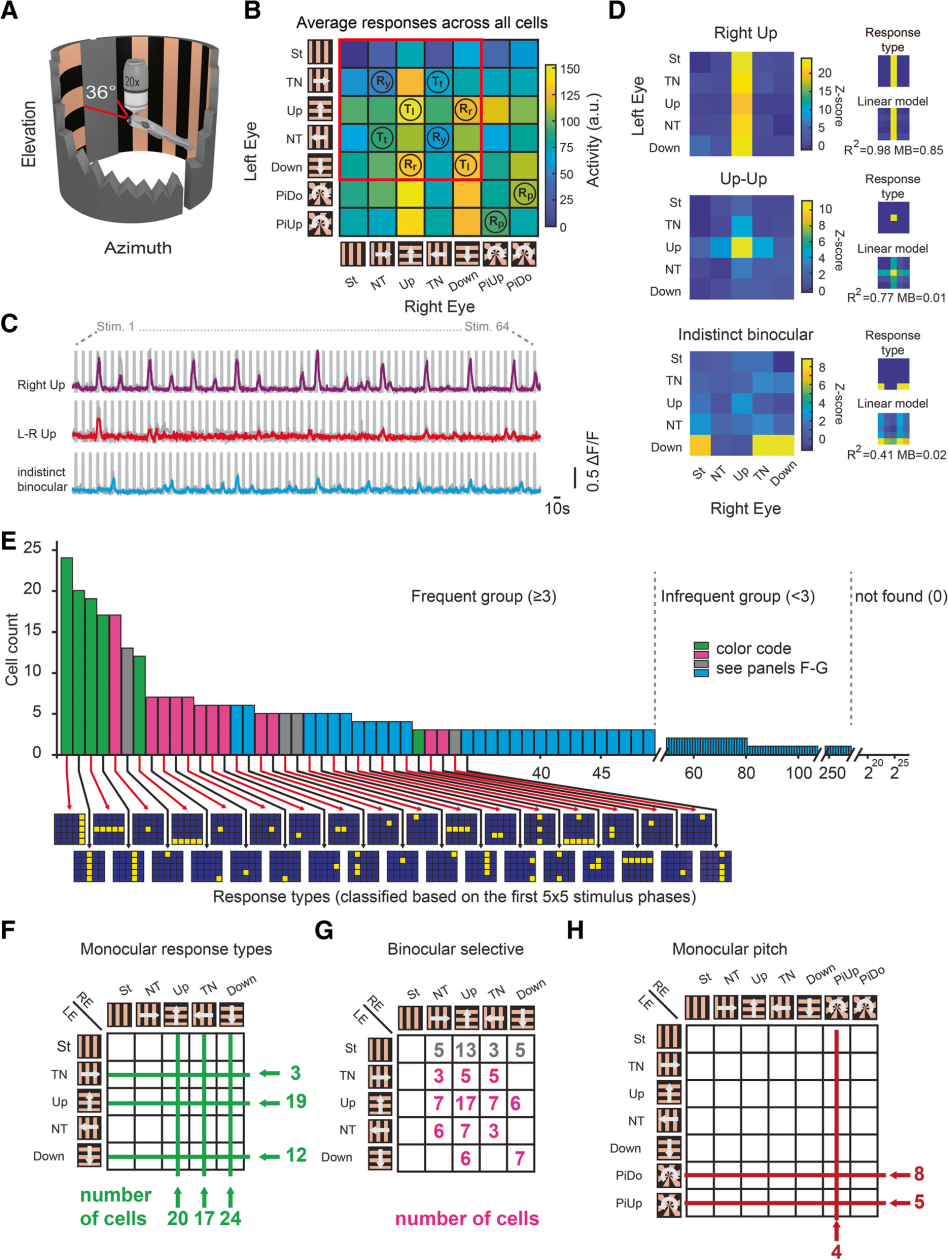
Binocular selective neurons in the optic tectum and pretectum. **a** Two half-cylindrical arenas were used to present moving gratings. The binocular zone (nasal 36°) was blocked. **b** The stimulus protocol consisted of 7 × 7 binocular motion phases. A unique combination of stimuli (stationary: St, temporal-to-nasal: TN, upwards: Up, nasal-to-temporal: NT, downwards: Down, pitch up: PiUp, pitch down: PiDo) was shown to both eyes in each phase. For each stimulus combination, the summed activity (z-score) across motion-sensitive neurons is indicated in arbitrary units (a.u.). The red rectangle indicates the 5 × 5 stimulus phases further analyzed in panels **d–g**. *R*_p_, *R*_r_, *R*_y_: binocular pitch, roll and yaw rotational stimulus phases. *T*_t_, *T*_l_: binocular thrust (forward/backward) and lift (up/down) translational stimulus phases (also see Fig. 4a). **c** Calcium responses of three example cells (median activity across three repetitions). The rectangular gray shades correspond to the 64 motion stimulus phases (Additional file 1: Figure S1E). **d** Calcium activity heat maps, classified binary response types and linear model fits of the cells from (**c**). **e** Binary response type analysis. The number of neurons (in pretectum and tectum, *n* = 8 animals, four composite brains, see “Materials and methods”) is plotted versus the ~ 34 million (2^25^) theoretically possible binary response types. Green, monocular response types; magenta and gray, binocular-selective response types; light blue: indistinct binocular response types. The first 34 frequent response types are illustrated below. Yellow, responsive phases; blue, non-responsive phases. **f** The number of neurons for the frequent monocular response types (each line corresponds to one response type), **g** binocular-selective response types (active only during one binocular stimulus combination) and **h** monocular pitch-responding type (only active during the indicated monocular pitch phases) are indicated in green (monocular responsive), magenta (binocular selective), gray (stationary selective), and red (pitch responsive). LE/RE: left/right eye

We have previously shown that many neurons of the zebrafish pretectum differentially respond to rotation and translation in the horizontal plane (clockwise, counterclockwise, forward, or backward motion). Many of these neurons were selectively tuned to one specific binocular optic flow pattern [10]. Based on the assumption that the binocular responses result from simple combinations of synaptic inputs from monocular neurons and the four monocularly preferred directions identified above, stimulus motion along the rostral-caudal and dorsal-ventral axes was used in the binocular direction selectivity experiment. To investigate the three-dimensional binocular encoding of optic flow in the zebrafish brain and how the monocular visual representations are combined binocularly, we designed seven monocular stimulus patterns consisting of (1) stationary grating (St), (2) temporal-to-nasal motion (TN), (3) nasal-to-temporal motion (NT), (4) up, (5) down, (6) pitch down, and (7) pitch up. Presentation of each of these seven stimuli to both eyes resulted in 7 × 7 = 49 combinations of binocular stimulus patterns (Fig. 2a, b).

For each neuron, we identified the stimulus patterns for which the neuron was active (see “Materials and methods”). We then classified neurons into binary response types based on the active/silent status for all stimulus phases (Fig. 2c, d). We analyzed responses to St, TN, NT, up, and down separately from the responses to pitch stimuli (see “Materials and methods”). The binarization of neuronal response profiles during the first 5 × 5 = 25 stimulus phases (TN, NT, up, down, and St) resulted in 2^25^ (ca. 34 million) mutually exclusive, theoretically possible response types. The number of frequent response types, however, for which three or more neurons (Fig. 2e) were identified was much smaller than this: 49. These 49 response types (excluding pitch-specific response types) accounted for a large proportion (39%) of the recorded motion-sensitive neurons (300/763 neurons). Within these 49 response types, about a third of the neurons (32%, 95/300) showed simple monocular DS responses without any contribution of motion presented to the other eye (Fig. 2d, top, and 2f). However, we also frequently observed binocular selective neurons, which were activated only during one specific combination of motion presented to the left and the right eye and silent to other combinations of motion (35%, 105/300, Fig. 2d, middle, and 2g). Among these binocular selective neurons, those coding for up-up (i.e., up to the right eye and up to the left eye), down-down, roll rightwards (down-up), roll leftwards (up-down), TN-TN (forward), and NT-NT (backward) were found frequently (5 to 17 neurons each). TN-NT and NT-TN neurons (yaw rotation-selective) were only found three times each. In our separate analysis of responses to pitch stimuli, we identified monocular pitch-responding neurons (Fig. 2h) and also four binocular pitch-selective neurons. Each of these pitch neurons was silent during the first 5 × 5 stimulus phases. Additional auxiliary analyses, which included all neurons and avoided forcing them into particular binary response types as above, confirmed the binocular selectivity of pretectal neurons (Additional files 1 and 2: Figures S1 and S2).

Taken together, our data show that a large fraction of the motion-sensitive pretectal and tectal neurons encode specific binocular optic flow patterns during horizontal, vertical, and pitch motion, resulting, among others, in translation- or rotation-selective responses in three different axes.

### Pretectal computations can distinguish between optic flow stimuli that elicit forward swimming and turning OMR behavior

**Fig. 3:**
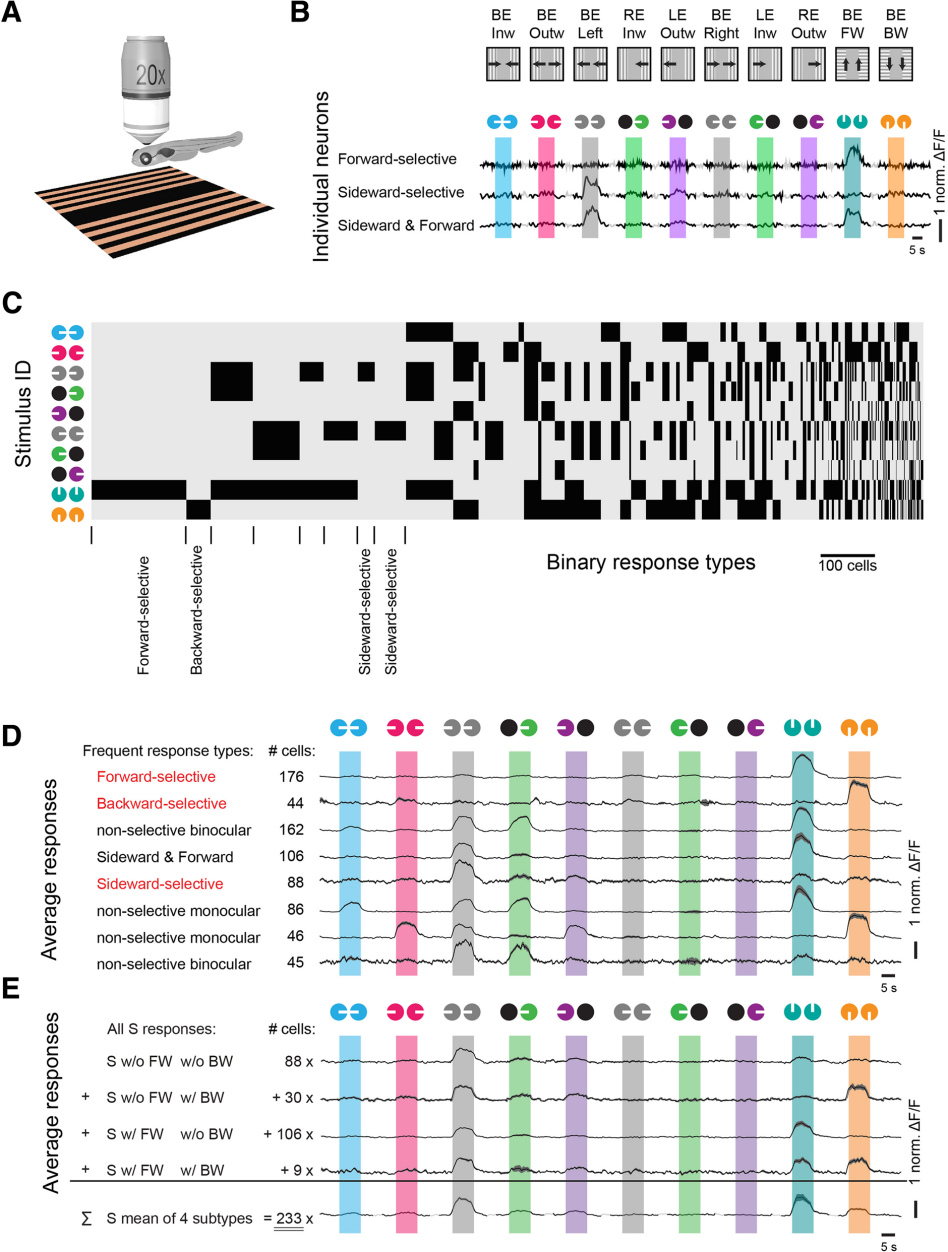
Pretectal binocular selective neurons processing visual stimuli presented from below. **a** The visual stimulus was presented from below the animal, while calcium activity was recorded from above. **b** *Top:* Ten binocular stimulus phases were repeated 3 to 10 times. The black arrowheads indicate the gratings’ moving directions. *Bottom*: normalized ΔF/F calcium responses for three examples, forward-selective, sideward-selective, and forward-and-sideward-selective neurons. White bars indicate the gratings’ moving directions for the corresponding eyes (colored circles); black circles represent eyes when stationary gratings were presented. **c** Neuronal response types were classified based on the calcium activity during the ten stimulation phases (*y*-axis, black: active). The width in the *x*-axis corresponds to the cell number for each response type. Response types are ordered according to frequency. **d** Response profiles of the eight most frequent response types (Additional file 3: Figure S3 for the mirror-symmetrical response types). Response types labeled “non-selective” are active for more than one stimulus phase. Response types labeled “binocular” are influenced by stimulus motion presented to either eye. **e** Grouped and averaged response profiles of all neurons (leftward or rightward) that are sideward-selective, when only the first 8 stimulus phases are considered, corresponding to response type “S” in the previous study [9]. The first four rows show all possible response combinations for S type cells (including all 10 — not just 8 — stimulus phases) and the last row shows the weighted average of all S type neurons. The cell numbers correspond to the sum of S type cells and the mirror-symmetrical S′ type cells. Note that the response type exclusively active for leftward motion (S w/o FW w/o BW, first row) cannot be detected when all responses are merged together (fifth row). In the study by Naumann et al., only this fifth merged response type has been reported

To investigate whether the same type of binocular selectivity also exists for stimulus combinations presented from below the animal, we analyzed a dataset from a previously published study [9] (Fig. 3a). The stimuli were chosen to elicit two different behaviors during certain stimulus phases: leftward/rightward OMR turning behavior was elicited by sideward visual motion and forward OMR swimming was elicited by forward visual motion [21, 22]. Five different monocular stimuli (leftward, rightward, forward, backward, and stationary gratings) were combined binocularly to achieve in total 10 different binocular stimulus phases (Fig. 3b, Inw, moving inward; Left, moving sideward to the left; Outw, outward; FW, forward; Right, moving to the right; RE, right eye, LE, left eye and BE, both eyes), resulting in 2^10^ = 1024 possible binary response types. However, as in the experiment in Fig. 2, only a very small fraction of the theoretically possible response types were detectable in the activity recordings (Fig. 3c–e and the response profiles of the mirror-symmetrical rightward counterparts in Additional file 3: Figure S3). Activity was generally phase-locked to motion (Fig. 3b, motion phases are indicated by the colored bars which share the same colors of the icons above) and the eight most frequent response types (and their six mirror-symmetrical counterparts) encompassed 45% (753/1672) of the neurons (Fig. 3d, the indicated neuron numbers correspond to the sums of the response type pairs active during leftward and rightward motion, except for FW and BW response types.).

The frequent response types fall into three major response categories. First, more than 13% (176 + 44 neurons) were selective for optic flow corresponding to forward or backward translation. Second, 5% of the recorded neurons (88/1672) were selective for optic flow corresponding to sideward translation. Third, the other neurons showed responses that were not fully selective for either forward, backward, or sideward translation, i.e., they were also active during additional binocular stimulus combinations.

## Discussion

**Fig. 4:**
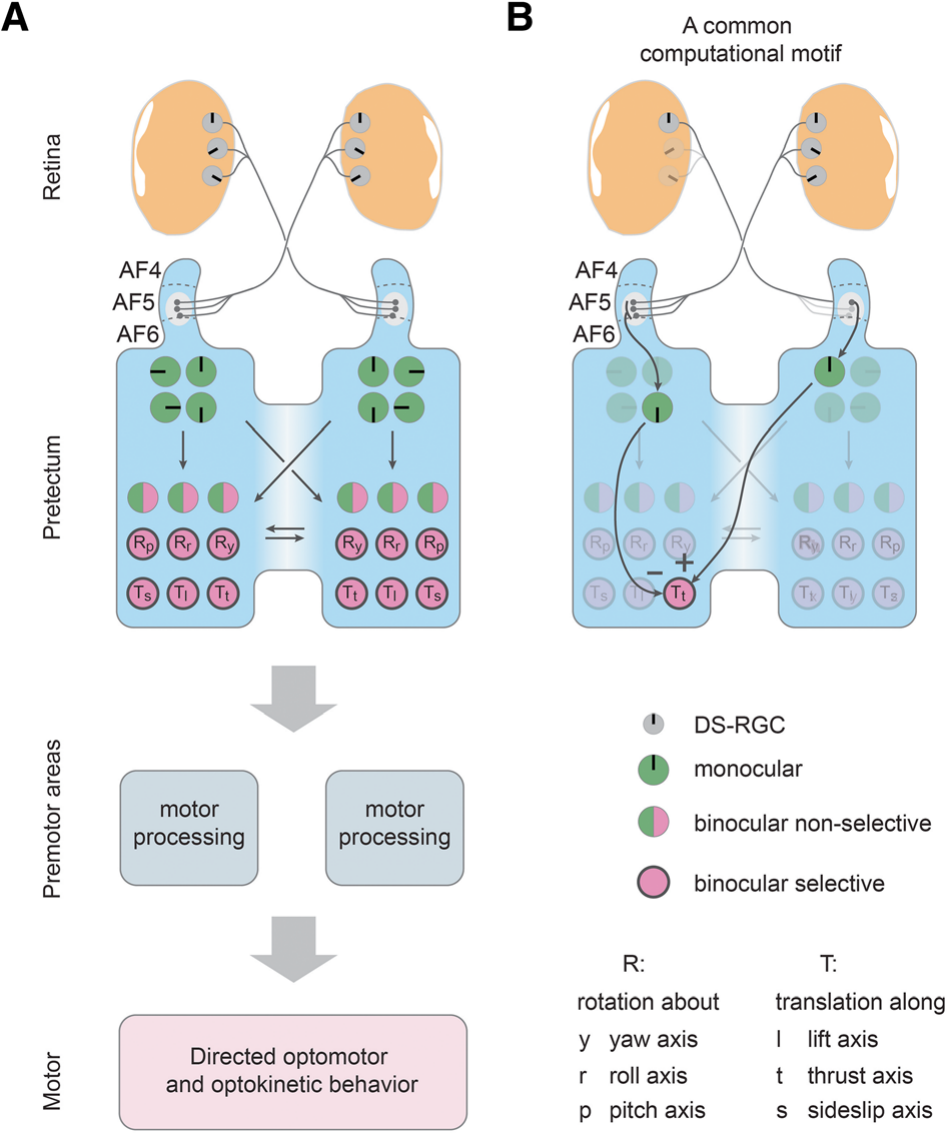
Proposed circuit model for binocular processing of optic flow in the pretectum. **a** The pretectum receives its DS-RGC inputs mainly via the pretectal arborization field AF5 (F. Kubo, personal communication, October 2018) and a transformation of represented motion axes occurs between retina (three PDs [14, 15]) and pretectum (four PDs). The monocularly responsive pretectal DS cells (green) send commissural projections (via the posterior commissure) to the contralateral pretectum, resulting in binocular non-selective response types (half green/half magenta) and binocular selective (magenta) response types. The binocular-selective neurons code for a single rotational or translational optic flow direction and could directly drive appropriate optomotor and optokinetic behavior via premotor structures such as the nucleus of the medial longitudinal fasciculus and oculomotor nuclei. *R*_p_, *R*_r_, *R*_y_: neurons selective for rotations about the pitch, roll and yaw axes, respectively. *T*_s_, *T*_t_, *T*_l_: neurons selective for translations along the sideslip (sidewards left/right), thrust (forward/backward), and lift (up/down) axes, respectively. Please note that only these six axes were tested in this study and it is possible that other (oblique) global optic flow axes result in even stronger responses, as suggested by previous reports on other species (see “Discussion”). **b** A proposed common computational motif shared across different planes of motion is antagonistic inputs from monocular pretectal neurons to binocular-selective neurons coding for motion in the same plane (here in the horizontal plane): A left eye monocular neuron in the right pretectal half excites the Forward-selective neuron (*T*_t_) during forward translation, while a right eye monocular neuron in the left pretectal half inhibits the selective neuron during nasal-to-temporal motion in the right eye, thus establishing forward selectivity

We show that both pretectum and optic tectum contain monocular DS neurons tuned to one out of four roughly orthogonally arranged directions. In addition, monocular pitch-selective neurons were also identified. This layout is in agreement with previous reports on the pretectum/AOS in other species [1, 19, 23]. Zebrafish swim in a three-dimensional environment. During forward swimming, they mainly experience horizontal motion, although pitch angle changes are associated with swimming bouts [24]. The perpendicularly arranged preferred directions are potentially the result of efficient coding strategies. The angular spacing between the neighboring represented preferred directions does, however, not match precisely 90° in each case, and this “odd” angular spacing could potentially correspond to a specific adaptation of the zebrafish visual system, which still remains to be discovered and understood. Since zebrafish DS-RGCs show different directional preferences than somata in the pretectum/tectum [15, 25], a transformation of directional axes needs to be computed in the pretectum/tectum [13]. Since the optic chiasm is completely crossed in the lateral-eyed zebrafish, pretectal binocular integration is likely established by the intra-pretectal commissures connecting the pretectum across hemispheres (Fig. 4a, b). In our experiment, we detected about 49 frequent response types. These responses encode different binocular combinations of image motion in each eye along the four cardinal directions.

Most of our manually drawn regions of interest (see “Materials and methods”) corresponded to the contours of individual cells (Additional file 4: Figure S4), confirming that the binocular responsivity described in this study was only minimally affected by potential cross-talk from multiple cells in our calcium imaging experiments. Many binocular neurons were selective for particular binocular combinations of visual stimuli that occur during translation, while being suppressed or non-responsive during any of the other combinations of binocular stimulus patterns. Furthermore, abundant sideward-selective responses were identified in the pretectum using stimuli presented from below (Fig. 3). Binocular rotational responses were also frequently observed, particularly for optic flow generated during roll or pitch, while yaw-specific responses were underrepresented. The number of identifiable binocular-selective neurons depends on the choice of the active/non-active threshold (Additional files 5 and 6: Figures S5 and S6). However, binocular-selective and monocular response types are still identifiable at each tested threshold, providing further support for our conclusions.

Our finding of abundant pretectal translation- and rotation-selective responses differs from findings reported in a recent study investigating zebrafish OMR circuits [9]. Since there were 10 stimulus phases, there were 2^10^ = 1024 theoretically possible response types. However, in the authors’ analysis, only the first eight stimuli (w/o forward and backward) were considered for building the response types, so that in their analysis the “sideward-selective” response type contained forward-responsive signals as well (see explicit example in Fig. 3e). This analysis difference explains why the forward translation-selective and sideward translation-selective pretectal responses have not been reported in the original study (see Fig. 5b in [9]).

The stimulus sets that were used in the experiments presented here were not fully realistic. For example, our forward “translation” stimulus (Fig. 2) did not contain realistic optic flow contraction in front of the animal as would be expected during backward ego-motion in a contrast-rich environment. Furthermore, some of the monocular motion stimuli (e.g., right eye inward in Fig. 3 and stimulus TN-St in Fig. 2) contained a stationary stimulus presented to one eye, which results in conflicting information for the fish. While presentation of such stimuli helps scientists to understand how the zebrafish brain processes motion, such stimuli are unlikely to occur in nature and therefore might evoke different (e.g. smaller) brain responses than more naturalistic stimuli would evoke.

Our results are in agreement with reports on binocular neurons in the pretectum/AOS of other species [26–28]. However, in birds and goldfish, the preferred axes for rotations and translations have been shown to differ from the motion axes used in our stimulus set. Three preferred axes have been reported, with one corresponding to the vertical axis (yaw rotation, lift translation) and two axes located in the horizontal plane, oriented at 45° to either side of the midline [16, 29–32]. Such layout supports a common reference frame with the axes of the vestibular canals. In this study, we intended to investigate the binocular combination of the four monocularly preferred directions, which roughly correspond to the Cartesian directions (see Fig. 1). We therefore did not include stimuli containing binocular motion along or about the horizontal oblique axes suggested in previous studies. If the binocular neurons are not tuned to oblique directions of motion in any part of their receptive fields (which seems likely given that during monocular stimulation the vertical or horizontal motion is preferred, see Fig. 1), then the precise preferred (binocular) rotation and translation axes largely depend on the receptive field locations. Suppose a hypothetical neuron with one small receptive field patch in each eye, responding to up in the right eye and down in the left eye. In total, this neuron has two receptive field patches, like the binocular bipartite receptive fields that have been measured in the rabbit AOS [33]. If the receptive field patches were located laterally in each eye, the neuron would respond maximally to stimulus rotations about the roll axis. If, however, the receptive field patches were located on the equator but shifted nasally (by 45° relative to the lateral location) for the right eye and temporally for the left eye, the neuron would prefer the horizontal axis identified in previous studies (+ 45° to the left from the midline). Since the receptive field sizes and centers have not been investigated in this study, more elaborate stimulus sets, including binocular oblique motion, would be necessary to identify the precise orientation of the preferred binocular motion axes for translation and rotation. This would allow to conclude whether the preferred motion axes in zebrafish correspond to those identified in other vertebrate species or not. Furthermore, knowledge about receptive field locations is needed to relate response types identified using sideward stimulation and stimulation from below. For example up-down binocular-selective neurons (Fig. 2) could correspond to sideward-selective neurons (Fig. 3), if the receptive fields of both eyes were located laterally and slightly below the equator of the view field.

We found that binocular responsive neurons are present in both pretectum and optic tectum. While the function of binocular neurons in the pretectum is likely related to its role in mediating OKR and OMR behavior (as discussed above and below), the role of binocular tectal neurons is less clear. While the optic tectum is dispensable for OKR and OMR in zebrafish [34], the tectal binocular neurons might still be involved in modulating these behaviors or be involved in different behaviors that require binocular representations. The high number of identified binocular response types (in comparison to the number of recorded neurons) made it difficult to compare binocular representations across the two brain areas. Ideally, the comparison of the binocular responsivity in these two brain areas should also include an estimation of the receptive field sizes in each eye, since pretectal receptive fields are expected to be larger than tectal ones. However, receptive field size estimation was not included in this study, since the associated long stimulus protocol durations prohibited it.

In the invertebrate visual system of flies, the optic flow is considered to be encoded by the lobula plate tangential cells, mainly including vertical system neurons (VS) and horizontal system neurons (HS), which are directional selective for vertical and horizontal motion, respectively [35, 36]. Intriguingly, these neurons show large and complex monocular or binocular receptive fields, with different preferred directions across local receptive field positions, resembling the roll rotational or nasalward translational optic flow [36–39]. Similarly, bipartite receptive fields with opposing preferred directions, e.g., upwards and downwards, within one entire receptive field of one neuron have been identified in the rabbit AOS and pigeon vestibulocerebellum, though these bipartite receptive fields do not precisely match any optic flow pattern expected to occur during ego-motion [33, 40].

Taken together, our experiments and analyses revealed the existence of binocular sensory neurons in pretectum and optic tectum which are selective for optic flow corresponding to motion in each of the six degrees of freedom (Fig. 4): rotation (roll, pitch, yaw) and translation (lift, thrust, sideslip). These selective neurons could—in principle—directly instruct appropriate OKR and OMR behavior, since no further sensory processing is needed. Indeed, in the case of OKR, direct projections of pretectal neurons into a cranial nerve nucleus responsible for horizontal eye movements (nucleus abducens) have been shown to exist in other vertebrate species [1, 18, 19].

A biologically faithful model, describing each processing step from the retina to OMR and OKR behavioral outputs, would significantly advance our understanding of neural processing principles in the vertebrate brain. Future studies are needed to investigate the connectivity of functionally identified cells in the pretectum, as well as the receptive field properties of DS-RGCs and pretectal neurons.

## Conclusion

The optic tectum and pretectum of larval zebrafish contain motion-sensitive neurons, which each prefer one out of four directions (up, down, temporal-to-nasal, nasal-to-temporal). Many neurons are binocular selective and respond maximally to either rotational or translational motion along or about one of three orthogonally arranged axes.

## Materials and methods

### Contact for reagents and resource sharing

Further information and requests for resources and reagents should be directed to the Contact Aristides Arrenberg (aristides.arrenberg@uni-tuebingen.de).

### Animal care and the transgenic line

All animal procedures conformed to the institutional guidelines of the University of Tübingen. The previously described transgenic zebrafish line *Tg(HuC:GCaMP5G)a4598Tg* was used in this study [20]. The transgenic line was kept in either a TL or TLN (nacre) background. Zebrafish larvae were raised in E3 medium until day 5 or 6 post-fertilization (dpf).

### Animal preparation

At the day of experiments (5 or 6 dpf), larvae were transferred into a petri dish and embedded in 1.6% low melting agarose (E3 medium). The agarose surrounding the eyes was not removed as to minimize the range of possible eye movements. Ten animals received an injection of α-bungarotoxin into the caudal vein to paralyze them and prevent eye movements and motion artifacts. Seven animals were recorded without paralysis (regarding the neuronal responses, we did not observe any differences between paralyzed and non-paralyzed animals and therefore we pooled the data from all fish). The animals were then transferred and mounted in agarose on a glass triangle and the fish head protruded the point of the glass triangle, so that the eyes could see through the (agarose and) water clearly. The volume of agarose surrounding the fish head was trimmed on the sides and in front of the animal in order to reduce the amount of agarose surrounding the eyes. However, the eyes were still covered by agarose to minimize the range of possible eye movements. The glass triangle was held from the back by a 5-mm-thin shaft which was fixed to an 8-cm-diameter glass bulb (made by a glass blower) filled with E3 medium. The glass bulb resembled a consumer market light bulb (threading of the light bulb/glass bulb shaft in the back of the fish), and a 5-cm-diameter hole was cut on the top of the spherical part to allow lowering the microscope objective towards the fish. From stimulus arena to the fish eye, the light traveled through air, glass (light hit glass roughly orthogonally in the spherical part as to minimize refraction of light rays), water, and finally agarose. The glass bulb was fixed with its shaft (15.5 mm diameter) to the metal holder, which allowed making adjustments in pitch and yaw (the glass shaft allowed for adjustments in roll). In the monocular direction selectivity experiments, the first batch of data (*n* = 5 larvae, included in the reported results) were acquired using a cylindrical plastic stage and container instead of the glass bulb container with glass triangle stage. In the first batches of pretectal monocular direction selectivity experiments (*n* = 5 larvae), we noticed a problem with reflections on the transparent stage wall on the opposite side of stimulation (plastic cylinder), resulting in responsive neurons on the ipsilateral side of (intended) stimulation in the tectum. In the second set of recordings (monocular DS: *n* = 4 larvae), we exclusively used the glass bulb stage and wrapped a piece of black, half-cylindrical aluminum foil around the objective. The black aluminum foil was then lowered beyond the eye contralateral to the stimulus in order to prevent this eye from seeing the reflections. In these recordings, only very few ipsilateral tectal neurons were detected. The ipsilateral neurons from the first batch (with reflection issue) are depicted in a more transparent color in Fig. 1g, while the ipsilateral neurons from the second batch have normal colors in Fig. 1g. Both sets of recordings were included in data analysis, because only few additional neurons were detected due to the reflections in the ipsilateral tectum, suggesting that the vast majority of the detected neurons of the contralateral side were detected due to the stimulus presented to the intended eye.

### Microscopy of somatic responses

Calcium imaging was performed with a two-photon microscopy setup based on the MOM microscope (Sutter Instruments, Novato, CA, USA) [41], using a Coherent Vision-S Ti-Sa laser and a × 20/1.0 Zeiss objective to image calcium signals in the transgenic fish line *HuC:GCaMP5G* (*Tg(elavl3:GCaMP5G)a4598*) [20]. Calcium time series were recorded at two frames per second, with an image size of 512 × 512 pixels and × 2 magnification, at 920 nm, pre-pulse compensation set to 9756 fs^2^. The midbrain and diencephalon were sampled from + 60 μm below the landmark (posterior commissure) to − 80 μm above the landmark. Optical slices were taken every 10 μm (monocular direction selectivity) or 20 μm (binocular stimulation) in the dorsoventral direction in individual fish. Across individual fish, all dorsoventral positions were recorded in 10-μm increments relative to the landmark (i.e., no recording at e.g. 5 or 15 μm below the landmark). We recorded eight animals using the binocular stimulus protocol, which corresponded to four recorded composite brains (one optical slice per 10 μm). Since we only recorded every 10 μm in dorsoventral extent for the monocular direction selectivity dataset, more than twice as many DS neurons should have been detectable in the respective brain areas (Fig. 1d), if we had sampled the brain areas at optimal spacing given the neuron soma diameter of ca. 5 μm. Where specified, error bars correspond to measures per completely imaged brain volume. Care was taken to record the same number of slices in each anatomical region. Oftentimes, more than one animal was used to image one brain volume completely. Due to the long recording times and positioning instability (likely resulting from the fish drifting within its agarose embedding), we corrected position drifts along the optical axis manually during the recording (mostly less than 4 μm per 30 min). Using the × 20 objective and a magnification of × 2, our spatial resolution was 0.43 μm/pixel on the *x*-axis (medial-lateral) and the *y*-axis (anterior-posterior).

### LED arena for visual stimulation (Figs. 1 and 2)

Visual stimulation of zebrafish was conducted with a cylindrical LED arena consisting of 14,336 LEDs (Kingbright TA08-81CGKWA): 2 (arena halves) × 8 (rows) × 14 (columns) × 64 (8 × 8 multiplexed LED matrix) LEDs. The caudal-most column of each arena half was left without LEDs (i.e., 14, not 15 columns), since the space was needed for the glass bulb stage metal holder. The arena covered − 168° to + 168° in azimuth and − 40° to 40° in elevation. A few degrees in angle of the dorsal field of view were likely blocked by the objective due to its access angle of 38.39° (< 40°); however, the eyes were located ~ 200 μm below the objective focus which should have resulted in a maximal viewing angle exceeding 38.39° (i.e., 39.2°). In the binocular stimulation experiments, the nasal view was intentionally blocked from − 18° to + 18° azimuth to prevent the stimulus being visible to the non-intended eye. The LEDs emitted at 570 nm and an additional high-pass filter foil (LEE no. 779, article 595-1700-7790, castinfo.de, Hagen, Germany) and diffusion filter foil (LEE no. 252, article 595-1780-2520) were placed in front of the arena to optimize GCaMP signal detection and make the stimulus appear more homogeneous. This resulted in a yellow appearance of the stimulus. The LED arena was controlled as described previously [42, 43]. LEDs were only lit during fly-back time of the scanning mirrors. Due to the high repetition rate of the line scans (~ 1000 Hz), the stimulus was perceived as continuous (not flickering) by the fish.

### Identification of regions of interest

For analysis of activity, a custom Matlab script (MOM_Load) identified regions based on their correlation to the stimulus (using a regressor that was active during motion stimuli and inactive otherwise) and ROIs were manually drawn as described previously [10, 44]. The 3D mapping of cell location was performed using custom written Matlab scripts (Midbrain Localizer and Cell Viewer), which allowed to register the two-dimensional recordings to a 3D z-stack which was acquired after recording sessions [10].

Since manual selection always comes with an inherent bias and potential confounds, we estimated the impact of the manual selection, on the monocular direction selectivity analysis and the binocular selectivity analysis, by first manually assigning three categories to each ROI, either (1) single-cell ROI, (2) multiple-cell ROI, or (3) unclear ROI (Additional file 4: Figure S4A). The vast majority of ROIs corresponded to the “single-cell” class, likely a result of our careful drawing of ROIs using information from the median image time series to visualize the outline of the cells. With this classification, we repeated the binocular direction selectivity analysis (Additional file 4: Figure S4B–D). We only found very few ROIs for which the (complex) response types could have been explained by potential overlap of signals from two neurons. Furthermore, no evidence for signal mixing was apparent when we plotted the linear model R^2^ and MB index values for the ROIs that corresponded to multiple cells (Additional file 7: Figure S7B, method see “Binocular directional tuning”). The distribution looked similar to the one for the single-cell ROIs (Additional file 7: Figure S7A), again supporting the validity of our manual ROI selection. Therefore, our post-hoc analysis suggests our manual ROI drawing only had minor impact on our conclusions.

To further test the potential impact and bias of the manual selection on binocular selectivity of the neurons, Pearson’s linear correlation coefficient between each pixel within a manually selected ROI and the average activity of the ROI for the binocular direction selectivity analysis data was calculated with Matlab. The histogram analyses of the average Pearson’s linear correlation coefficients from the identified monocular and binocular neurons or single-cell and multiple-cell ROIs were compared, respectively. The average correlation coefficient in binocular neurons was lower than that in monocular neurons (Additional file 7: Figure S7D). There are three possible explanations. First, monocular neurons are much more active with our stimulus protocol, while binocular neurons typically only show activities to one or a few stimulus phases, leading to a lower signal-to-noise level and pixel-wise correlation in these binocular neurons. Second, binocular-selective neurons (which correspond to a large fraction of the binocular neurons plotted in Additional file 7: Figure S7D) are defined by their selective responses: they do not fire in a pattern that resembles a superposition of two monocular-responsive neurons, but rather fire only when stimuli were presented to both eyes (for one particular stimulus phase). Therefore, it is quite unlikely that binocular-selective ROIs would erroneously result from monocular neuronal responses (but of course, the non-linearity of the calcium indicator needs to be considered). A third possible reason for the higher pixel-wise correlation values of monocular neurons could be that the monocular neurons are more frequent in the optic tectum, which is located superficially in the animal, resulting in a higher signal-to-noise ratio for tectal recordings. As expected, the average correlation coefficient of isolated ROIs (single-cell ROI) was about the same as that of the potentially contaminated ROIs (multiple-cell ROI, Additional file 7: Figure S7E).

In addition, another analysis based on principal component analysis and clustering was performed (Additional file 8: Figure S8). To show the potential of the technique, we first used four ROIs identified as separate cells in the manual selection. Principal component analysis (PCA) and subsequent expectation maximization clustering were performed for all pixels of the four ROIs. To identify the optimal number of clusters, we used the Bayesian information criterion (BIC). We then used the clusters and can show that the method is well suited to separate non-correlated sources, even when they are in close proximity (Additional file 8: Figure S8A), because pixels from the same ROIs clustered together. The same technique was applied to find the optimal number of clusters (i.e., different neurons) in the ROI masks that were previously classified as most likely being composed of multiple sources (Additional file 8: Figure S8B). As the analyses described above already suggested, the resulting fluorescence pixel time series showed high correlation with each other for individual ROI masks (Additional file 8: Figure S8C), suggesting that the variability stems mostly from noise and not from separated sources that were erroneously assumed to be one cell.

### 3D anatomical mapping

To distinguish pretectal from tectal neurons, we proceeded as follows. For each fish, the whole z-stack—which was imaged from the top of tectum to deep ventral pretectum—was resliced to generate a transverse view in each image plane. On selected, regularly spaced transverse planes (more than ~ 50 planes), the ventral border of the tectum was drawn: on each of these transverse plane (512 pixels from left to right, *x* dimension), a curve was drawn through the area devoid of neuronal somata and fluorescence, which was ventrally adjacent to periventricular tectal area with densely packed, fluorescent somata. From each curve, 51 homogeneously distributed points were selected as key points with which a new boundary curve was generated by linear interpolation or three-term Gaussian fitting. Using this method, we obtained a boundary curve with 512 data points corresponding to the pixels in *x*/*y* dimensions (left-right and dorsal-ventral) for each transverse plane (Additional file 1: Figure S1A, curves in red color). In-between the annotated transverse planes, the 2D curves were interpolated to receive a surface that separated the tectum from the pretectum in all three dimensions.

### Monocular directional tuning

We presented moving gratings in eight directions (48°/s) to the right eye of the fish with one LED half-cylindrical arena. In order to control for possible adaptation of neural activities, we showed the visual stimuli in two different orders for different individual animals as depicted in Additional file 1: Figure S1B. All eight visual stimulus phases were repeated three times. A stationary grating (pause) was presented between motion phases, as illustrated in Additional file 1: Figure S1E. The spatial frequency of the visual stimuli was 0.033 cycles/°. Each stimulus period started with a stationary grating (4 s), and then the motion stimulus phase (using the same, but moving grating) ensued for 4.8 s. After each motion phase, the stationary pattern was presented for 2 s (pause) and then the pattern was changed to the stationary grating of the next stimulus period (4 s). Tectal and pretectal identity was assigned to neurons based on the semi-automatically drawn anatomical border visible in the z-stack (see 3D anatomical mapping). Neurons below the tectum and more rostral than 140 μm caudal of the landmark (anterior edge of the posterior commissure, see Kubo et al. [10]) were considered as pretectal neurons and plotted in the histogram of preferred directions. Neurons located more caudally (> 140 μm caudal to the landmark) potentially corresponded to premotor neurons (nMLF) and were therefore excluded from further analysis.

Motion phase-locked activity for individual neurons was calculated by a series of analysis steps. First, we calculated a DFF (ΔF/F) trace from the raw fluorescence trace [DFF = (*F*(*t*) − *F*_b_)/*F*_b_, with *F*_b_ corresponding to the baseline fluorescence]. Then, we filtered the DFF fluorescence traces with a low pass wavelet decomposition [type Daubechies, Matlab: wavedec (DFF,1,‘db4’) and a sliding median filter (the median of three data points). Then, deconvolution was performed on the filtered data with the decay time constant (tau) of GCaMP5G, 1.5 s. We calculated the mean of phase-averaged signal (MPAS; averaged over stimulus phase time) from the deconvolved traces. The baseline was defined as the MPAS of all the non-stimulus phases (i.e., without moving stimulus). The standard deviation (STD) of all phase-averaged signals was calculated for the non-motion phases. And the z-score was calculated using the equation [z-score = (MPAS − mean (baseline))/STD (baseline)]. We then calculated the median MPAS z-score (i.e., the median across the three repetitions of a stimulus phase of the average of all data points within one stimulus phase).

The tuning curve of the calcium signal z-score from eight directions was fitted with one or two von-Mises distributions (Matlab function 1: A/(2π × besseli (0, κ)) × exp.(κ × cos(X − PD)) + baseline, function 2: A1/(2π × besseli (0, κ)) × exp.(κ × cos(X − PO)) + A2/(2π × besseli (0, κ)) × exp.(κ × cos(X – PO − π)) + baseline). In the well fitted data (*R*^2^ > 0.8), the preferred direction (PD), the response (*R*_pref_) in the PD, and the opposite direction (*R*_opp_) were calculated from the fitting with one von-Mises distribution. Similarly, we calculated the preferred orientation (PO) of the motion (not the grating), the response in the PO (*R*_ori_) and the orthogonal orientation (*R*_orth_) from the fitting with the sum of two von-Mises distributions which had peaks separated by 180°. The DSI and OSI were calculated as: DSI = (*R*_pref_ − *R*_opp_)/(*R*_pref_ + *R*_opp_) and OSI = (*R*_ori_ − *R*_orth_)/(*R*_ori_ + R_orth_). The neurons with DSI larger than the threshold; 0.7 were considered as direction selective. The following criteria were used to define orientation-selective neurons: OSI > 0.5 and DSI < 0.7. In the histogram analysis of the PDs for tectal and pretectal neurons, 6° was used as the bin width. We identified the peaks of the PDs via fitting the histogram data with the sum of four von-Mises distributions (plus a baseline summand). For each of the four peaks *i*, the summand took the following form: *a*_i_ × exp.(κ × cos(X − Peak_i_))/(2π × besseli (0, κ_i_)).

The agarose embedding resulted in a slightly variable pitch orientation of the body across animals. The magnitude of the pitch rotation was between 1° and 10° for each animal. Pitch correction of the PD values did not result in sharper tuning curves. We present the PD data with pitch correction for body positions here. Note that we did not measure torsional eye positions and it is therefore possible that eyes had different pitch positions relative to the body.

To calculate the laterality index, we took the number of cells contralateral to the stimulated eye and subtracted the number of ipsilaterally located neurons and divided the result by the sum of neurons in both hemispheres. This resulted in an index running from 1 (all contralateral) to − 1 (all ipsilateral).

### Binocular directional tuning

In the binocular DS experiments, moving bars or rotating radial patterns, at the velocity of 15°/s, were used as visual stimuli. The spatial frequency was 0.067 cycles/°. We showed 3 repetitions of the 64 combinatory stimulus phases using two half-cylindrical stimulus arenas. The combinations were shown in a pseudo-randomized order, as illustrated in the Additional file 1: Figure S1E (in the diagonal direction shown by the numbers and the first few phases are indicated by red arrows). Patterns from both arenas were changed during the pause phase to temporally separate the possibly occurring calcium responses to a changed static stimulus from the motion-sensitive calcium responses we were interested in. In each trial, every stimulus phase lasted 4.8 s, followed by a 6 s pause. During the first 2 s of the pause, the stimulus combination remained on the arenas and for the remaining 4 s the next stimulus combination was presented motionlessly. The repetitions were separated by 9-s long pauses. In a pilot experiment, we recorded neural activity in two animals with a stimulus protocol consisting of 12 × 8 instead of 8 × 8 stimulus phases. In the additional phases, one of the eyes was stimulated with 45°/135°/225°/315° oblique motion directions. We only found very few neurons that preferred binocular stimulus combinations including oblique motion or had specifically suppressed activity during oblique motion. This result indicated that oblique directions apparently do not play a major role in binocular optic flow representations, which was in agreement with our results from monocular stimulations. We therefore decided to omit the oblique stimulus phases in the stimulus protocol used to acquire the data presented here.

Tectal or pretectal identity was assigned to neurons based on the semi-automatically drawn anatomical border visible in the z-stack (see section on “3D anatomical mapping”). Neurons below the tectum that were not located in the brain volumes corresponding to nMLF, vEMN, and dVEMN (see gray brain volumes in Fig. 1g) were considered as pretectal neurons and included in further analysis, while the neurons from (pre-) motor brain volumes were excluded.

To analyze the directional tuning of neurons, we reduced the complexity by using a binary response type (RT) classification [9, 10]. To do this, we compared the average inter-stimulus interval before every stimulus phase with the median averaged activity in the stimulus phase. If the mean ΔF/F during the stimulation exceeded 3 × STD over its local baseline, it was assigned a 1, otherwise a 0. This method was applied to all stimulus phases (7 × 7 + 1), excluding stimulus phases that included a blank screen on one of the half cylinders. We excluded these 14 (7 + 7) phases after noticing that the reflections of about 4% of the light on the inside of the opposite glass bulb wall (theoretically to be expected, see Fresnel equations) were visible to the fish eye which should have received a black stimulus during this stimulation phase. In the calcium imaging data, we could clearly see that many (but not all) neurons responded to the motion of the reflected stimulus as well. Since the activity during these 14 stimulus phases was therefore contaminated, we decided to remove those phases from further analysis. In all other phases (except the black-black phase), a bright stimulus was presented on both halves of the arena so that the reflection was likely hard to see for the animal and did not confound the analysis of the rest of the data. A potential caveat of the binary classification of motion phases in active and inactive is that the selectivity of cells depends on the chosen threshold. A low threshold will introduce noise to the binary classification, while a high threshold will lead to increased “selectivity” of cells that might still respond to other stimulation phases, but whose responses do not reach the threshold to be classified as active. In order to confirm the validity of the chosen threshold, we performed an additional analysis, in which a lower (2.5 × STD + mean) and a higher (3.5 × STD + mean) threshold were used. This analysis returned similar results as that of using the 3 × STD threshold and therefore suggests that our chosen threshold value is an acceptable threshold, which did not strongly bias response types to getting classified as binocular selective or monocular responsive (Additional file 6: Figure S6).

Additionally, we calculated the “MB index,” a metric that depends on the fit of measured activity to a linear model consisting of purely monocular responses (Fig. 2d and Additional file 1: Figure S1F). To examine how well the neuronal responses can be explained by monocular direction-selective excitatory input, we fitted a linear model to the data recorded with the binocular stimulus protocol and calculated the goodness of fit *R*^2^ value. The coefficients of the model were used to calculate the monocular-binocular index (MB index) for each neuron. The function consists of 14 summands, each a product of a weight (α for columns, β for rows) and a 7 × 7 matrix consisting of one column or row with ones (C and R in the depicted linear model equation). The neural activity (z-scores) of a given neuron during the 7 × 7 stimulus phases (i.e., 49 z-score values) is then fitted to the equation to identify how well the neuronal responses can be explained by monocular direction-selective excitatory input, which should always activate a whole row or column. To calculate the MB index, we took the difference between the sum of all row coefficients and the sum of all column coefficients and divided it by the sum of all coefficients for normalization. A value close to 1 or − 1 corresponds to mainly monocularly driven neurons, while a value of 0 corresponds to binocularly driven neurons. The expectation would be that cells whose activity can be explained by input from excitatory DS-RGCs in the absence of inhibition (e.g., monocularly driven simple cells) would have a high *R*^2^ value and additionally a high (absolute) MB index value, since they should be active only in one row or column of the 7 × 7 matrix (i.e., their activity depends on the stimuli presented to a single eye, while the other eye’s stimulus does not affect activity). The activity of many neurons could not be explained by this simple linear regression model, while the model worked well for monocular neurons. This difference indicates that the activity of the former neurons strongly depended on the combination of binocular phases, i.e., these neurons performed binocular computations. Note, that the “MB index” analysis of response profiles did not depend on thresholds or classifications and still assessed whether the activity of a neuron in question can be explained by input from DS-RGCs alone or shows evidence of binocular inhibitory computations. The MB index helps to visualize the grade of binocular response selectivity and binocular drive. Low values of the goodness of fit to a linear model (*R*^2^) on the *x*-axis in the scatter plot (Additional file 1: Figure S1G) correspond to neurons with binocular selectivity. High values can be interpreted as neural responses without suppressed activity during particular binocular phases. In the case of TN, up, NT, and down (but not pitch) responses, neurons with high *R*^2^ can be fully explained by simple excitatory input from DS-RGCs (monocular and binocular simple responses). The MB index is plotted on the *y*-axis in Additional file 1: Figure S1G. Red points correspond to cells shown in Fig. 2d.

We subdivided all further analysis to reduce the theoretical complexity of response types (RTs), which is given by 2^49^ possible RT combinations and the receptive field (RF) locations of neurons. Our Pitch stimuli were different from the remaining motion phases in that the motion directions differed wildly across the surface of the stimulus arena. Since we did not know the RF locations in these experiments, it was difficult to interpret the neuronal activity observed during Pitch stimulation. For example, a neuron that is responding to Up in the right eye and also to Pitch-Up could be non-specific for Pitch and simply have a relatively small RF in the nasal visual field at the level of the equator. However, it is also possible that this neuron could have a large receptive field and a complex selectivity for RE Up and RE Pitch-Up. Without proper knowledge about the RF locations, in an RT classification based on all 2^49^ possible RTs, the mere locations of (small) RFs will already result in several different found response types (combinations of Pitch responses with other motion responses). Furthermore, the limited recording time prohibited a combination of RF mapping and binocular directional selectivity analysis of a sufficiently high number of animals and recordings to identify all such response types at a sufficient frequency. We therefore analyzed Pitch responses separately from the other stimulation phases. We selected the five phases of each eye corresponding to stationary (ST), temporal-to-nasal (TN), Up, nasal-to-temporal (NT), and Down, leading to a total of 25 stimulation phases. Based on these 25 phases we then classified neurons, which had the same binary response type. The same analysis was applied to stationary and pitch stimulation phases alone. Pitch and stationary phase RTs were further sub-selected to exclude overlap with the 5 × 5 analysis (i.e., not active during the 5 × 5 phases for the pitch analysis and not active during the 6 × 6 motion phases for the stationary analysis). The resulting RTs were then classified as either frequent (≥ 3 cells) or infrequent RTs (≤ 2 cells).

While 39% of the neurons contributed to the group of the frequent response types, for the large number of infrequent response types, only one or two neurons were identified. It was unclear whether these neurons just had noisy response profiles and thereby largely resembled the more frequent response types, or whether these neurons showed a very special responsiveness. To estimate the potential variability and the possibility of missing important RTs, we calculated the distance of infrequent to frequent RTs by first identifying the most similar frequent RT to an infrequent response based on the difference in the binary RT profile from the 5 × 5 analysis (Additional file 1: Figure S1H) for each neuron. For this purpose, we calculated the correlation of its response profile (5 × 5 z-score heat map as in Fig. 2d) with the median response profile of the best matching response type from the frequent group (red line). We used Pearson correlation of the median ΔF/F response of all neurons within the best-fitting frequent RT with the ΔF/F response of the infrequent response type in question. We further calculated the correlation of the individual responses of neurons in a frequent RT group with its own RT median response (blue line in Additional file 1: Figure S1H). As a control, we calculated the correlation of each neuronal response to a randomly selected existing RT (random choice of an existing RT response, drawn from a distribution accounting for the frequency of each RT). We then plotted the cumulative sum of neurons for each of the three analyses (individual frequent RT vs. RT median, infrequent individual RT vs. most similar RT median, any neuron vs. random RT median) versus the achieved Pearson correlation (Additional file 1: Figure S1H). This analysis was performed to show the validity of the approach and the assignment of binary RTs. Note that the responses of the neurons from the infrequent group were mostly very similar to one of the more frequent response types, suggesting that the computational repertoire (in the context of the employed stimulus phases) is well described by the first 49 most frequent response types.

The binocular-selective response types presented in Fig. 2 are only active during one particular stimulus phase (a combination of two motion directions in the left and right eye, respectively) and silent during all other stimulus phases. These neurons clearly show the needed selectivity to detect a particular type of optic flow, e.g., to distinguish rotational from translational optic flow. However, other neurons that are active during two or more stimulus phases can still be somewhat binocular selective for certain directions (and maybe not selective for certain other directions). In order to characterize the existence of selectivity in single neurons that is suited to distinguish rotation from translation in the same plane of motion (e.g., forward versus clockwise for stimuli moving in the horizontal plane), we devised a “phase pair analysis” (Additional file 2: Figure S2). We compared responses to 18 different stimulus phase pairs (BW-CC, FW-CC, FW-BW, CW-CC, CW-BW, CW-FW, RCC-TD, RCW-TD, RCW-RCC, TU-TD, TU-RCC, TU-RCW, PU-AP2, PD-AP2, PD-PU, AP1-AP2, AP1-PU, AP1-PD; forward, FW; backward, BW; clockwise, CW; counterclockwise, CC; roll clockwise, RCW; roll counterclockwise, RCC; translation up, TU; translation down, TD; pitch up, PU; pitch down, PD; antagonistic pitch directions 1 (PiUp-PiDo), AP1; antagonistic pitch directions 2 (PiDo-PiUp), AP2.) and classified neuronal responses to phase pairs in four different categories (1–1, 1–0, 0–1, 0–0). If cells are generally able to distinguish between these different types of motion (rotation vs. translation), the expectation would be that we have an over-proportionally large number of 0–1 or 1–0 classifications. For instance, with our visual stimuli, motion could occur in the horizontal plane, transversal plane or sagittal plane (stimulus phase fields without filled text in Additional file 2: Figure S2A correspond to optic flow combinations that should not occur frequently in nature and have therefore not been analyzed here). For each kind of motion in the three planes, there are four stimulus phases. The six possibilities of combining two of these four phases for the horizontal plane of motion are depicted in Additional file 2: Figure S2A. For each of the 3 × 6 = 18 possible stimulus phase pairs, there are four possible response types for each neuron, which are illustrated for the possible stimulus phase pair CW-FW: active during both stimulus phases (1:1), selective for phase 1 or selective for phase 2 (1:0, and 0:1), or the neuron can remain silent during these two stimulus phases (0:0). We analyzed the responses of all 591 motion-sensitive neurons for the presence of super-threshold activity in the 3 × 4 stimulus phases of interest and calculated how frequent the four different phase pair responses were for each of the 3 × 6 = 18 stimulus phase pairs in question. Every neuron contributes a count of 1 to just one out of the possible four binary combinations (1:1, 1:0, 0:1, 0:0), according to its active/silent status for the stimulus phase pair in question. The *y*-axis in Additional file 2: Figure S2A is labeled “number of observations” and not “number of neurons,” because we analyzed the responses of all 591 neurons for each of the 18 phase pair combinations. Since virtually all neurons were only active for a small fraction of the 64 stimulus phases from the original stimulus protocol, the (0:0) phase pair responses are very frequent. The monocular, simple neurons should be responsible for many of the observed (1:1) phase pair responses. The (1:0) and (0:1) phase pair responses can be inspected in this plot to judge how selective the neurons were for particular types of optic flow directions. For example, selective responses to rotation (CW, CCW) appeared to be less frequent than responses to translation (FW, BW) in the horizontal plane (Additional file 2: Figure S2B). We performed a bootstrap analysis to verify which of the selectivities were significantly over- or underrepresented than the chance level. For each motion plane, we calculated the percentage of neurons that responded during 0, 1, 2, 3, or 4 stimulus phases out of the available four binocular stimulus phases. We then simulated response types of 591 neurons by drawing the number of active stimulus phases (0 to 4) for each simulated neuron and randomly specifying which of the four phases are active. This was done for each plane of motion separately and 25000 simulated datasets of 591 neurons were generated. The simulated data was then processed in the phase pair analysis as described above. The percentage of datasets, in which a higher or lower number of neurons was found for each of the bars, was determined in order to achieve a one-tailed *p* value (bootstrapping, alpha = 0.05) and the results are plotted (Additional file 2: Figure S2C). To account for multiple comparisons, we also performed Bonferroni correction, which led to a threshold of < 0.00069 equalling 17 or fewer than 17 events (Additional file 2: Figure S2D).

### Inclusion criteria for somatic calcium responses

We calculated the Pearson linear correlation coefficients between the stimulus phase z-scores (see above) of the three stimulus protocol repetitions (for all three pairwise combinations) to characterize the reproducibility of stimulus-evoked calcium responses. In our further data analysis, we only kept the neurons for which all three correlation coefficients were higher than a certain threshold. The threshold was set between 0.7 and 0.8 to exclude around 20% neurons with low reproducibility of stimulus-evoked activity in the monocular direction selectivity experiment (2556 out of 3271, 78% neurons were kept). The thresholds for the binocular direction selectivity experiments were relatively lower, ranging from 0.65 to 0.75, due to the long experimental protocols and the low fraction of stimulus phases that a neuron was responsive to. Only 64% (853 out of 1325) neurons were kept for further analysis in the binocular direction selectivity experiments. We then performed signal-to-noise ratio (SNR) analysis to exclude neurons with unstable baseline. In the SNR analysis, a threshold of four was used on the z-score to detect positive neural responses. Signal-to-noise ratio (SNR) was defined as the ratio of the average response of all the responsive phases to the standard deviation of the baseline. All the neurons with SNR lower than a certain threshold were excluded. The threshold was set between 8 and 10 to exclude about 10% remaining neurons with SNR in the monocular direction selectivity experiment (2290 out of 2556, 90% neurons were kept). We kept about 90% (763 out of 853) neurons in the binocular direction selectivity experiments for further analysis.

### Analysis of the data from Naumann et al.

The data used for the pretectal forward/backward and sideward selectivity analysis were kindly provided by Florian Engert and Eva Naumann. The file contained (alongside with other information) repetition-averaged calcium responses for each neuron. Each stimulus phase response consisted of 64 data points each (including 19 data points before onset of motion, 3 data points per second). In addition, the file included the assigned binary classification (response type). We analyzed the neurons which had been stimulated with all 10 relevant stimuli (1672 pretectal neurons out of the 3070 neurons from Fig. 5B in their study). The DFF fluorescence traces were filtered with a low pass wavelet decomposition using a custom script [type Daubechies, Matlab: wavedec (DFF,1,‘db4’)]. The resulting traces were filtered again using a sliding median filter (the median of three data points). Then, the filtered data were deconvolved with the decay time constant (tau) of GCaMP5G, 1.5 s. The mean of stimulus phase-averaged signal calculated from the deconvolved traces was compared with an arbitrary threshold, the 1.0 × STD above the baseline of the deconvolved traces. To ensure that the chosen threshold reflects the underlying selectivity of neurons, we performed the same analysis with three different thresholds (1.0 × STD, 1.8 × STD, and 3 × STD + mean) and found that all major conclusions were still supported, irrespective of the chosen threshold (Additional file 6: Figure S6). The baseline was determined by taking the time periods without motion stimulus for each cell and calculating the mean of the response during each pause. The standard deviation was then calculated by taking the standard deviation of these mean values across stimulus pauses for each neuron. The value “1” was assigned to a stimulus phase when the mean value was larger than the threshold. Otherwise, the stimulus phase was assigned as the value “0.” In our analysis, all ten different stimulus phases, including the forward and backward motions were considered and theoretically 1024 (2^10^) different response combination classes could have been detected.

### Quantification and statistical analysis

The statistical information is provided in each of the sections above.

After obtaining the raw data (pixel calcium signal time series), the data was quantified, anatomically registered, and statistically analyzed according to the descriptions given in the above sections entitled “Identification of regions of interest”, “Monocular directional tuning,” “Binocular directional tuning,” and “Analysis of the data from Naumann et al.”.

The analyzed number of zebrafish and brains is indicated in the main text and figure legends. Error bars correspond to SEM unless stated otherwise.

## Additional files

Additional file 1: **Figure S1.** (related to Figs. 1 and 2). Experimental parameters and auxiliary analyses. (A) Defining the tectal-pretectal boundary. Each image is a transverse view and the number indicates the pixel position (1 pixel = 0.43 μm in the anterior-posterior direction). D, dorsal; V, ventral; R, right; L, left. (B) Monocular stimulus protocols to map preferred directions. Left: motion directions; Right: Temporal sequence (rightwards) of the two stimulus protocols used in different recordings. A, anterior; P, posterior. (C) Histogram of direction selectivity from all recorded motion-sensitive cells. (D) Histogram of the normalized vector sum of DS neurons (*n* = 9 brains, pretectum and tectum combined in (C) and (D)). (E) Stimulus protocol for the binocular direction selectivity experiment. Top: 8 × 8 unique stimulation phases were presented in the indicated order and repeated three times in the protocol. Bottom: Schematic of two individual stimulus periods. (F) Linear model equation used to assess functional properties of binocular optic flow processing (see “Materials and methods”). (G) Linear model analysis. Monocular neurons (with high absolute MB index) tended to be fit very well by the linear sum model, while more binocularly driven neurons (MB index close to 0) were not fit well. This suggests that binocularly driven neurons are often suppressed during particular stimulus phases, but not for other stimulus phases in the same row or column, thus establishing binocular response selectivity. (H) Analysis of the similarity of infrequent responses to the more frequent response types found in Fig. 2e. The red and blue lines show the cumulative distributions of the correlations of neurons from the infrequent group with the best matching frequent response type and correlations of neurons from the frequent group with their response type, respectively. The yellow line shows the correlation of the neuronal responses with a randomly selected, existing response type (shuffled). (JPG 10220 kb)

Additional file 2: **Figure S2.** (related to Fig. 2). Analysis of stimulus phase pairs for horizontal, transversal and sagittal planes of motion. (A) *Left*: possible binocular combinations of motion in the horizontal plane (gray text), transversal plane (orange), or sagittal plane (blue). *Middle*: For each kind of motion in the 3 planes, there are 4 stimulus phases. The 6 pair combinations of these phases are depicted for the motion in the horizontal plane. *Right*: For each of the 3 × 6 = 18 possible stimulus phase pairs, there are 4 possible response types for each neuron, which are illustrated for the possible stimulus phase pair CW-FW: active during both stimulus phases (1:1), selective for phase 1 or selective for phase 2 (1:0, and 0:1), or silent (0:0). (B) Phase pair responses across all 591 motion-sensitive neurons. The monocular, simple neurons should be responsible for many of the observed (1:1) phase pair responses. The (1:0) and (0:1) phase pair responses can be inspected in this plot to judge how selective the neurons were for particular types of optic flow directions, e.g., selective responses to rotation (CW, CCW) appeared to be less frequent than responses to translation (FW, BW) in the horizontal plane (compare the cyan and green bar heights). (C) Bootstrap analysis. “Higher” in red color denotes response types which were found significantly more frequently in the zebrafish brain than expected by chance in the shuffled dataset. (D) Same analysis as in (C), but using a two-tailed *p* value and Bonferroni correction for multiple tests (*n* = 72). The FW-selective responses were significantly more frequent than CC-selective responses for the FW-CC phase pair in column 2. For many phase pairs consisting of antagonistic directions, a significantly lower number of neurons with non-direction-selective 1:1 responses was identified (RCW-RCC, TU-TD, PD-PU), when compared to the shuffled data. (JPG 5187 kb)

Additional file 3: **Figure S3.** (related to Fig. 3). Analysis of pretectal response types (forward/backward and sideward). (A) Average response profiles of the eight most frequent response types (*Top*) and their (rightward-responding) mirror-symmetrical response types *(Bottom*). Except for FW and BW response types, for which no mirror-symmetrical response types exist, neuron numbers on the left correspond to the sums of the response type pairs active during leftward motion and their mirror-symmetrical counterpart. Numbers on the right correspond to the individual (non-merged) response types. (JPG 2301 kb)

Additional file 4: **Figure S4.** (related to Fig. 2). The manually drawn ROIs correspond to single neurons in most cases in the binocular stimulation dataset. (A) Examples of the manually drawn ROIs from the binocular direction selectivity analysis experiment. Single-cell ROIs (90% of all ROIs), multiple-cell ROIs (7%) and unclear ROIs (3%) are shown in three rows. For each example ROI, the left plot shows the manually selected ROI (in red) on the median fluorescence of the calcium signal time series. The z-score heat map is shown on the right plot. The warm color and blue color (from red to yellow, the correlation coefficient decreases) indicate the region where the fluorescence is correlated or reverse-correlated with the motion-stationary regressor (see Methods). The numbers in black indicated the neuron ID when we analyzed the data. (B) Binary response type analysis of the single-cell ROIs. The number of ROIs (in pretectum and tectum, *n* = 8 animals, 4 composite brains, see “Materials and methods”) corresponding to single-cell ROIs is plotted versus the ~ 34 million (2^25^) theoretically possible binary response types. The color code corresponds to monocular (green) and binocular selective neurons (magenta: selective for a single binocular motion stimulus, gray: selective for a single binocular stimulus containing motion on one side and a stationary grating on the other side; light blue: indistinct binocular response types). The first 34 frequent response types are illustrated below. Yellow, responsive phases; Blue, non-responsive phases. (C) Binary response type analysis of the multiple-cell ROIs, similar to (A). (D) Binary response type analysis of the unclear ROIs, similar to (A). (JPG 7743 kb)

Additional file 5: **Figure S5.** (related to Fig. 2). Binary response type analysis with different thresholds. (A) Response type analysis using 10 different thresholds. While the median number of identified neurons per response type is affected by the choice of threshold, the number of identified binocular-selective and monocular response types is only mildly affected by threshold choice. (B) A lower threshold of 2.5 × STD + mean was used. (C) Analysis for the original threshold of 3 × STD + mean. (D) Analysis using a higher threshold of 3.5 × STD + mean. STD and mean correspond to the standard deviation and mean of the calcium signal ΔF/F during the stationary phases (related to Fig. 2e). (JPG 7371 kb)

Additional file 6: **Figure S6.** (related to Fig. 3). Effect of changing the activity threshold on identified response profiles of the neurons from the data of Naumann et al. Response profiles of the eight most frequent response types are illustrated in (A, B) using different thresholds. The visual stimulus-evoked calcium signals were detected with thresholds, 1 × STD + mean (A), 1.8 × STD + mean (B) and 3 × STD + mean (C). In this study we used a threshold of 1 × STD, while the previous study used 1.8 × STD. Except for FW and BW response types, in each panel, the indicated neuron numbers on the left side of the plot correspond to the sums of the response type pairs active during leftward and rightward motion. On the right side of the plot, the numbers indicated the neuron numbers of each individual response type (i.e., without mirror-symmetrical response type). The mirror-symmetrical response type pairs were plotted separately on the upper and lower panel. The proportions of the forward-, backward-, and sideward-selective neurons are indicated on the right of each panel. STD, standard deviation of the calcium signal ΔF/F during the stationary phases; mean, mean of the calcium signal ΔF/F during the stationary phases. The icons and colors are identical to those in Fig. 3. (JPG 6249 kb)

Additional file 7: **Figure S7.** (Related to Fig. 2). Pixel-wise correlation analysis suggests that manually drawn ROIs correspond to single neurons in most cases in the binocular stimulation dataset. (A–C) Linear model analysis of the binocular experiment data for the single-cell ROIs (A), multiple-cell ROIs (B) and unclear ROIs (C) (related to Additional file 1: Figure S1G). (D) Histogram of average ROI correlation coefficients for the correlation between a given ROI’s pixel time series and the average fluorescence time series of this ROI, for single-cell ROIs from the 49 frequent response types (left, original plot; right, normalized plot). Blue, binocular neurons (binocular selective or indistinct neurons); pale red, monocular neurons. (E) histogram analysis of the correlation coefficients of the pixel time series to the average fluorescence of single-cell ROIs and multiple-cell ROIs from the 49 frequent response types (left, original plot; right, normalized plot). (JPG 5536 kb)

Additional file 8: **Figure S8.** (Related to Fig. 2). Clustering of local pixel correlations reveals highly correlated activity patterns for cells that were classified as multiple ROIs. (A) Examples demonstrating the effectiveness of the method. Four ROIs that were manually identified to be single cells (their activity traces are shown on the right) were combined and each pixel of these ROIs was correlated with each other. Subsequently, we performed principal component analysis (PCA) and expectation maximization clustering, which automatically segmented even spatially close neurons as independent units. Pixels were plotted according to the first two principal components (PCs) at the bottom, illustrating that that the developed algorithm (PCA and clustering) successfully identified the correspondence of the pixels to their original ROIs (lower left: color code based on manual ROI selection, lower right: color code based on assigned cluster identity). (B) Example cluster analysis showing a potential “multiple-cell” ROI (see manual analysis in Additional file 7: Figure S7) that was split into three separate clusters by the algorithm (left); and the accompanying activity traces for each cluster (shown on the right). (C) Example cluster analysis showing an ROI that wasn’t split, and its corresponding activity trace. (D) Quantification of the average correlation of the mean ROI traces resulting from the clustering (e.g., average correlation of the three traces shown in (B)). Correlation is overall high, suggesting that there is no major signal contamination even for neurons that were manually assigned to potentially contain multiple-cell activity. (JPG 2823 kb)

## References

[1] Masseck OA, Hoffmann KP (2009). Comparative neurobiology of the optokinetic reflex. Ann N Y Acad Sci.

[2] Orger MB, Gahtan E, Muto A, Page-McCaw P, Smear MC, Baier H (2004). Behavioral screening assays in zebrafish. Methods Cell Biol.

[3] Maaswinkel H, Li L (2003). Spatio-temporal frequency characteristics of the optomotor response in zebrafish. Vis Res.

[4] Busch C, Borst A, Mauss AS (2018). Bi-directional control of walking behavior by horizontal optic flow sensors. Curr Biol.

[5] Ibbotson MR, Hung YS, Meffin H, Boeddeker N, Srinivasan MV (2017). Neural basis of forward flight control and landing in honeybees. Sci Rep.

[6] Kretschmer F, Tariq M, Chatila W, Wu B, Badea TC (2017). Comparison of optomotor and optokinetic reflexes in mice. J Neurophysiol.

[7] Cazin L, Precht W, Lannou J (1980). Pathways mediating optokinetic responses of vestibular nucleus neurons in the rat. Pflugers Arch.

[8] Schiff D, Cohen B, Raphan T (1988). Nystagmus induced by stimulation of the nucleus of the optic tract in the monkey. Exp Brain Res.

[9] Naumann EA, Fitzgerald JE, Dunn TW, Rihel J, Sompolinsky H, Engert F (2016). From whole-brain data to functional circuit models: the zebrafish optomotor response. Cell..

[10] Kubo F, Hablitzel B, Dal Maschio M, Driever W, Baier H, Arrenberg AB (2014). Functional architecture of an optic flow-responsive area that drives horizontal eye movements in zebrafish. Neuron..

[11] Gahtan E, Tanger P, Baier H (2005). Visual prey capture in larval zebrafish is controlled by identified reticulospinal neurons downstream of the tectum. J Neurosci.

[12] Gabriel JP, Trivedi CA, Maurer CM, Ryu S, Bollmann JH (2012). Layer-specific targeting of direction-selective neurons in the zebrafish optic tectum. Neuron..

[13] Abbas F, Triplett MA, Goodhill GJ, Meyer MP (2017). A three-layer network model of direction selective circuits in the optic tectum. Front Neural Circuits.

[14] Hunter PR, Lowe AS, Thompson ID, Meyer MP (2013). Emergent properties of the optic tectum revealed by population analysis of direction and orientation selectivity. J Neurosci.

[15] Lowe AS, Nikolaou N, Hunter PR, Thompson ID, Meyer MP (2013). A systems-based dissection of retinal inputs to the zebrafish tectum reveals different rules for different functional classes during development. J Neurosci.

[16] Simpson JI, Leonard CS, Soodak RE (1988). The accessory optic system. Analyzer of self-motion. Ann N Y Acad Sci.

[17] Crowder NA, Wylie DR (2002). Responses of optokinetic neurons in the pretectum and accessory optic system of the pigeon to large-field plaids. J Comp Physiol A Neuroethol Sens Neural Behav Physiol..

[18] Cochran SL, Dieringer N, Precht W (1984). Basic optokinetic-ocular reflex pathways in the frog. J Neurosci.

[19] Masseck OA, Hoffmann KP (2008). Responses to moving visual stimuli in pretectal neurons of the small-spotted dogfish (Scyliorhinus canicula). J Neurophysiol.

[20] Ahrens MB, Orger MB, Robson DN, Li JM, Keller PJ (2013). Whole-brain functional imaging at cellular resolution using light-sheet microscopy. Nat Methods.

[21] Orger MB, Kampff AR, Severi KE, Bollmann JH, Engert F (2008). Control of visually guided behavior by distinct populations of spinal projection neurons. Nat Neurosci.

[22] Antinucci P, Hindges R (2016). A crystal-clear zebrafish for in vivo imaging. Sci Rep.

[23] Klar M, Hoffmann KP (2002). Visual direction-selective neurons in the pretectum of the rainbow trout. Brain Res Bull.

[24] Ehrlich DE, Schoppik D (2017). Control of movement initiation underlies the development of balance. Curr Biol.

[25] Nikolaou N, Lowe AS, Walker AS, Abbas F, Hunter PR, Thompson ID (2012). Parametric functional maps of visual inputs to the tectum. Neuron..

[26] Manteuffel G (1984). Electrophysiology and anatomy of direction-specific pretectal units in Salamandra salamandra. Exp Brain Res.

[27] Manteuffel G (1987). Binocular afferents to the salamander pretectum mediate rotation sensitivity of cells selective for visual background motions. Brain Res.

[28] Wylie DR (2000). Binocular neurons in the nucleus lentiformis mesencephali in pigeons: responses to translational and rotational optic flowfields. Neurosci Lett.

[29] Wylie DR, Bischof WF, Frost BJ (1998). Common reference frame for neural coding of translational and rotational optic flow. Nature..

[30] Simpson JI (1984). The accessory optic system. Annu Rev Neurosci.

[31] Wylie DR, Frost BJ (1993). Responses of pigeon vestibulocerebellar neurons to optokinetic stimulation. II. The 3-dimensional reference frame of rotation neurons in the flocculus. J Neurophysiol.

[32] Masseck OA, Hoffmann KP (2009). Question of reference frames: visual direction-selective neurons in the accessory optic system of goldfish. J Neurophysiol.

[33] Simpson JI, Leonard CS, Soodak RE (1988). The accessory optic system of rabbit. II. Spatial organization of direction selectivity. J Neurophysiol.

[34] Roeser T, Baier H (2003). Visuomotor behaviors in larval zebrafish after GFP-guided laser ablation of the optic tectum. J Neurosci.

[35] Krapp HG, Hengstenberg R, Egelhaaf M (2001). Binocular contributions to optic flow processing in the fly visual system. J Neurophysiol.

[36] Egelhaaf M, Kern R, Krapp HG, Kretzberg J, Kurtz R, Warzecha AK (2002). Neural encoding of behaviourally relevant visual-motion information in the fly. Trends Neurosci.

[37] Krapp HG, Hengstenberg R (1996). Estimation of self-motion by optic flow processing in single visual interneurons. Nature..

[38] Hopp E, Borst A, Haag J (2014). Subcellular mapping of dendritic activity in optic flow processing neurons. J Comp Physiol A Neuroethol Sens Neural Behav Physiol.

[39] Haikala V, Joesch M, Borst A, Mauss AS (2013). Optogenetic control of fly optomotor responses. J Neurosci.

[40] Winship IR, Wylie DR (2006). Receptive-field structure of optic flow responsive Purkinje cells in the vestibulocerebellum of pigeons. Vis Neurosci.

[41] Euler T, Hausselt SE, Margolis DJ, Breuninger T, Castell X, Detwiler PB (2009). Eyecup scope--optical recordings of light stimulus-evoked fluorescence signals in the retina. Pflugers Arch.

[42] Joesch M, Plett J, Borst A, Reiff DF (2008). Response properties of motion-sensitive visual interneurons in the lobula plate of Drosophila melanogaster. Curr Biol.

[43] Reiser MB, Dickinson MH (2008). A modular display system for insect behavioral neuroscience. J Neurosci Methods.

[44] Miri A, Daie K, Burdine RD, Aksay E, Tank DW (2011). Regression-based identification of behavior-encoding neurons during large-scale optical imaging of neural activity at cellular resolution. J Neurophysiol.

