# Supplementary figures and images for: Selective processing of all rotational and translational optic flow directions in the zebrafish pretectum and tectum
Source: BMC Biol. 2019 Mar 29;17:29. doi: 10.1186/s12915-019-0648-2 (PMC6441171; doi:10.1186/s12915-019-0648-2)

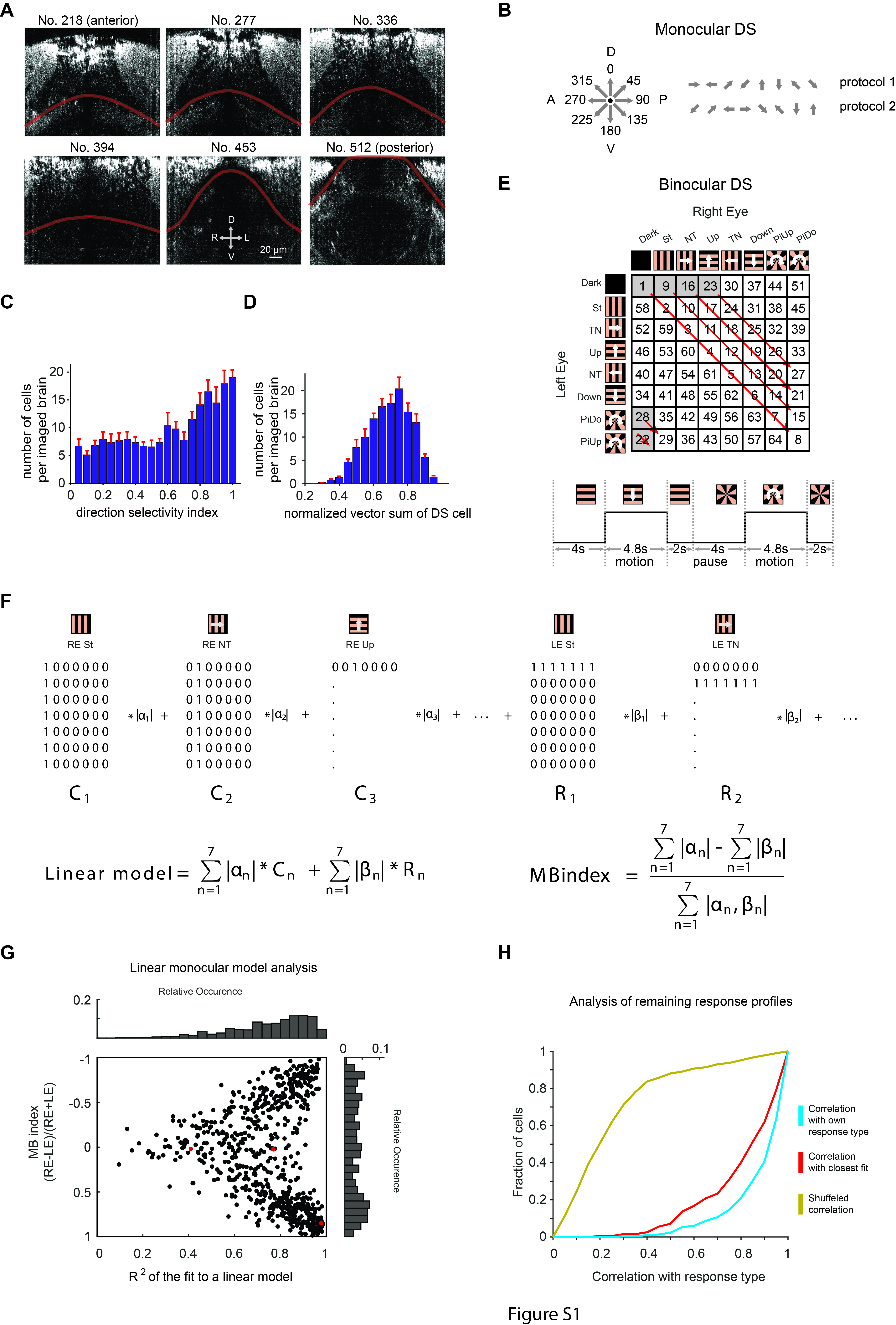

Supplement: Supplementary file 1 — Figure S1. (related to Figs. 1 and 2). Experimental parameters and auxiliary analyses. (A) Defining the tectal-pretectal boundary. Each image is a transverse view and the number indicates the pixel position (1 pixel = 0.43 μm in the anterior-posterior direction). D, dorsal; V, ventral; R, right; L, left. (B) Monocular stimulus protocols to map preferred directions. Left: motion directions; Right: Temporal sequence (rightwards) of the two stimulus protocols used in different recordings. A, anterior; P, posterior. (C) Histogram of direction selectivity from all recorded motion-sensitive cells. (D) Histogram of the normalized vector sum of DS neurons (n = 9 brains, pretectum and tectum combined in (C) and (D)). (E) Stimulus protocol for the binocular direction selectivity experiment. Top: 8 × 8 unique stimulation phases were presented in the indicated order and repeated three times in the protocol. Bottom: Schematic of two individual stimulus periods. (F) Linear model equation used to assess functional properties of binocular optic flow processing (see “Materials and methods”). (G) Linear model analysis. Monocular neurons (with high absolute MB index) tended to be fit very well by the linear sum model, while more binocularly driven neurons (MB index close to 0) were not fit well. This suggests that binocularly driven neurons are often suppressed during particular stimulus phases, but not for other stimulus phases in the same row or column, thus establishing binocular response selectivity. (H) Analysis of the similarity of infrequent responses to the more frequent response types found in Fig. 2e. The red and blue lines show the cumulative distributions of the correlations of neurons from the infrequent group with the best matching frequent response type and correlations of neurons from the frequent group with their response type, respectively. The yellow line shows the correlation of the neuronal responses with a randomly selected, existing response type (shuffled). (JP [file 12915_2019_648_MOESM1_ESM.jpg]

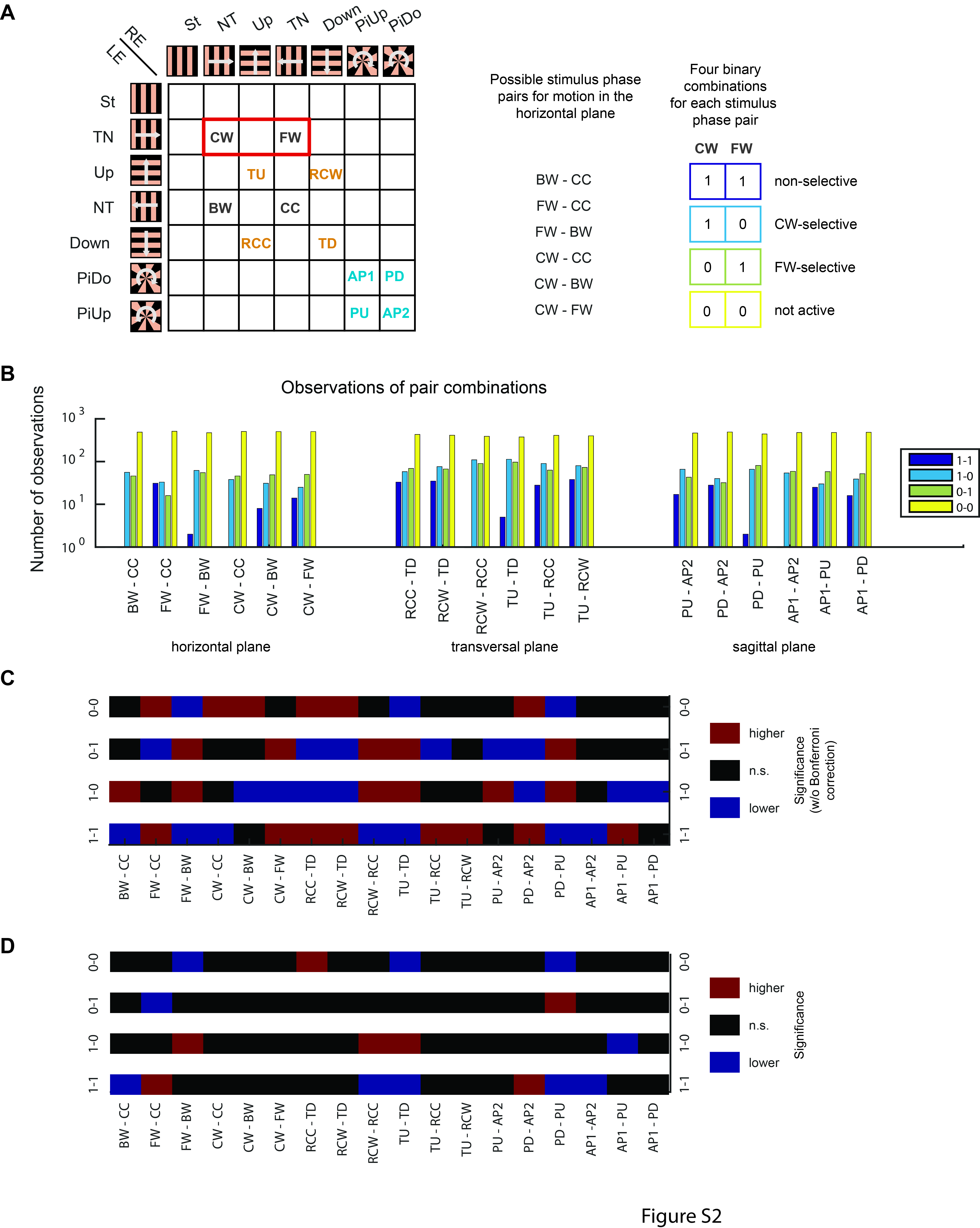

Supplement: Supplementary file 2 — Figure S2. (related to Fig. 2). Analysis of stimulus phase pairs for horizontal, transversal and sagittal planes of motion. (A) Left: possible binocular combinations of motion in the horizontal plane (gray text), transversal plane (orange), or sagittal plane (blue). Middle: For each kind of motion in the 3 planes, there are 4 stimulus phases. The 6 pair combinations of these phases are depicted for the motion in the horizontal plane. Right: For each of the 3 × 6 = 18 possible stimulus phase pairs, there are 4 possible response types for each neuron, which are illustrated for the possible stimulus phase pair CW-FW: active during both stimulus phases (1:1), selective for phase 1 or selective for phase 2 (1:0, and 0:1), or silent (0:0). (B) Phase pair responses across all 591 motion-sensitive neurons. The monocular, simple neurons should be responsible for many of the observed (1:1) phase pair responses. The (1:0) and (0:1) phase pair responses can be inspected in this plot to judge how selective the neurons were for particular types of optic flow directions, e.g., selective responses to rotation (CW, CCW) appeared to be less frequent than responses to translation (FW, BW) in the horizontal plane (compare the cyan and green bar heights). (C) Bootstrap analysis. “Higher” in red color denotes response types which were found significantly more frequently in the zebrafish brain than expected by chance in the shuffled dataset. (D) Same analysis as in (C), but using a two-tailed p value and Bonferroni correction for multiple tests (n = 72). The FW-selective responses were significantly more frequent than CC-selective responses for the FW-CC phase pair in column 2. For many phase pairs consisting of antagonistic directions, a significantly lower number of neurons with non-direction-selective 1:1 responses was identified (RCW-RCC, TU-TD, PD-PU), when compared to the shuffled data. (JPG 5187 kb) [file 12915_2019_648_MOESM2_ESM.jpg]

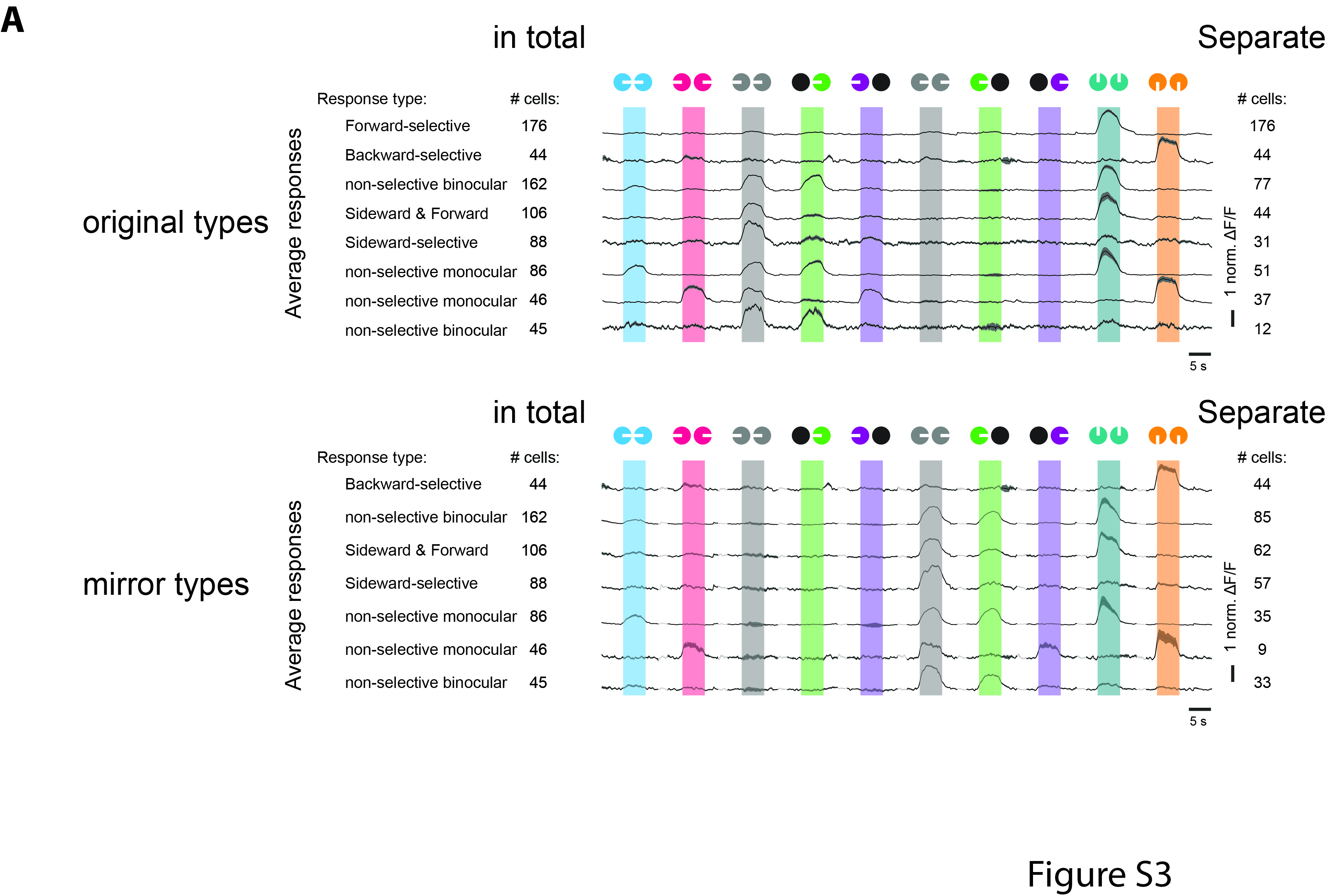

Supplement: Supplementary file 3 — Figure S3. (related to Fig. 3). Analysis of pretectal response types (forward/backward and sideward). (A) Average response profiles of the eight most frequent response types (Top) and their (rightward-responding) mirror-symmetrical response types (Bottom). Except for FW and BW response types, for which no mirror-symmetrical response types exist, neuron numbers on the left correspond to the sums of the response type pairs active during leftward motion and their mirror-symmetrical counterpart. Numbers on the right correspond to the individual (non-merged) response types. (JPG 2301 kb) [file 12915_2019_648_MOESM3_ESM.jpg]

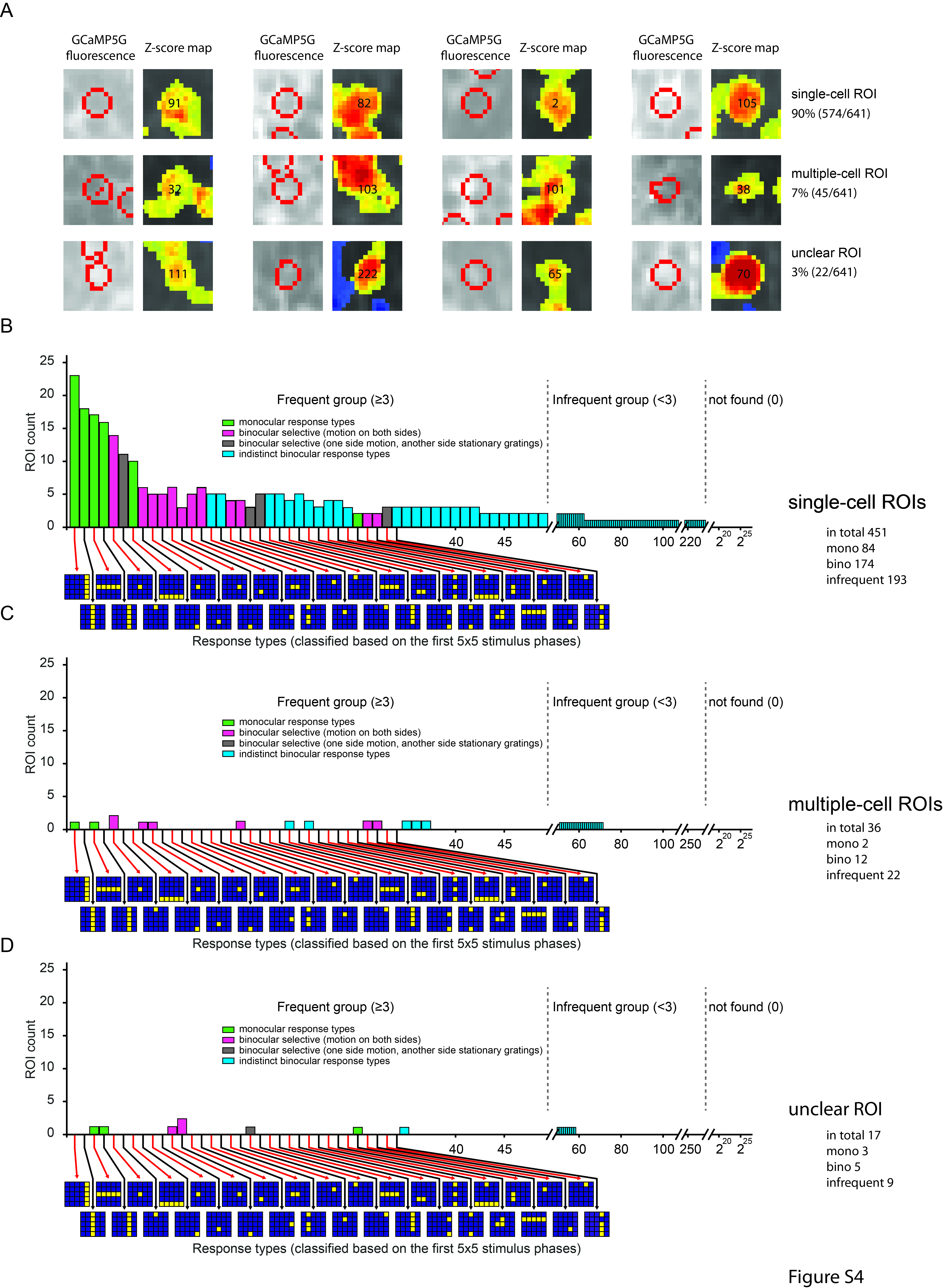

Supplement: Supplementary file 4 — Figure S4. (related to Fig. 2). The manually drawn ROIs correspond to single neurons in most cases in the binocular stimulation dataset. (A) Examples of the manually drawn ROIs from the binocular direction selectivity analysis experiment. Single-cell ROIs (90% of all ROIs), multiple-cell ROIs (7%) and unclear ROIs (3%) are shown in three rows. For each example ROI, the left plot shows the manually selected ROI (in red) on the median fluorescence of the calcium signal time series. The z-score heat map is shown on the right plot. The warm color and blue color (from red to yellow, the correlation coefficient decreases) indicate the region where the fluorescence is correlated or reverse-correlated with the motion-stationary regressor (see Methods). The numbers in black indicated the neuron ID when we analyzed the data. (B) Binary response type analysis of the single-cell ROIs. The number of ROIs (in pretectum and tectum, n = 8 animals, 4 composite brains, see “Materials and methods”) corresponding to single-cell ROIs is plotted versus the ~ 34 million (225) theoretically possible binary response types. The color code corresponds to monocular (green) and binocular selective neurons (magenta: selective for a single binocular motion stimulus, gray: selective for a single binocular stimulus containing motion on one side and a stationary grating on the other side; light blue: indistinct binocular response types). The first 34 frequent response types are illustrated below. Yellow, responsive phases; Blue, non-responsive phases. (C) Binary response type analysis of the multiple-cell ROIs, similar to (A). (D) Binary response type analysis of the unclear ROIs, similar to (A). (JPG 7743 kb) [file 12915_2019_648_MOESM4_ESM.jpg]

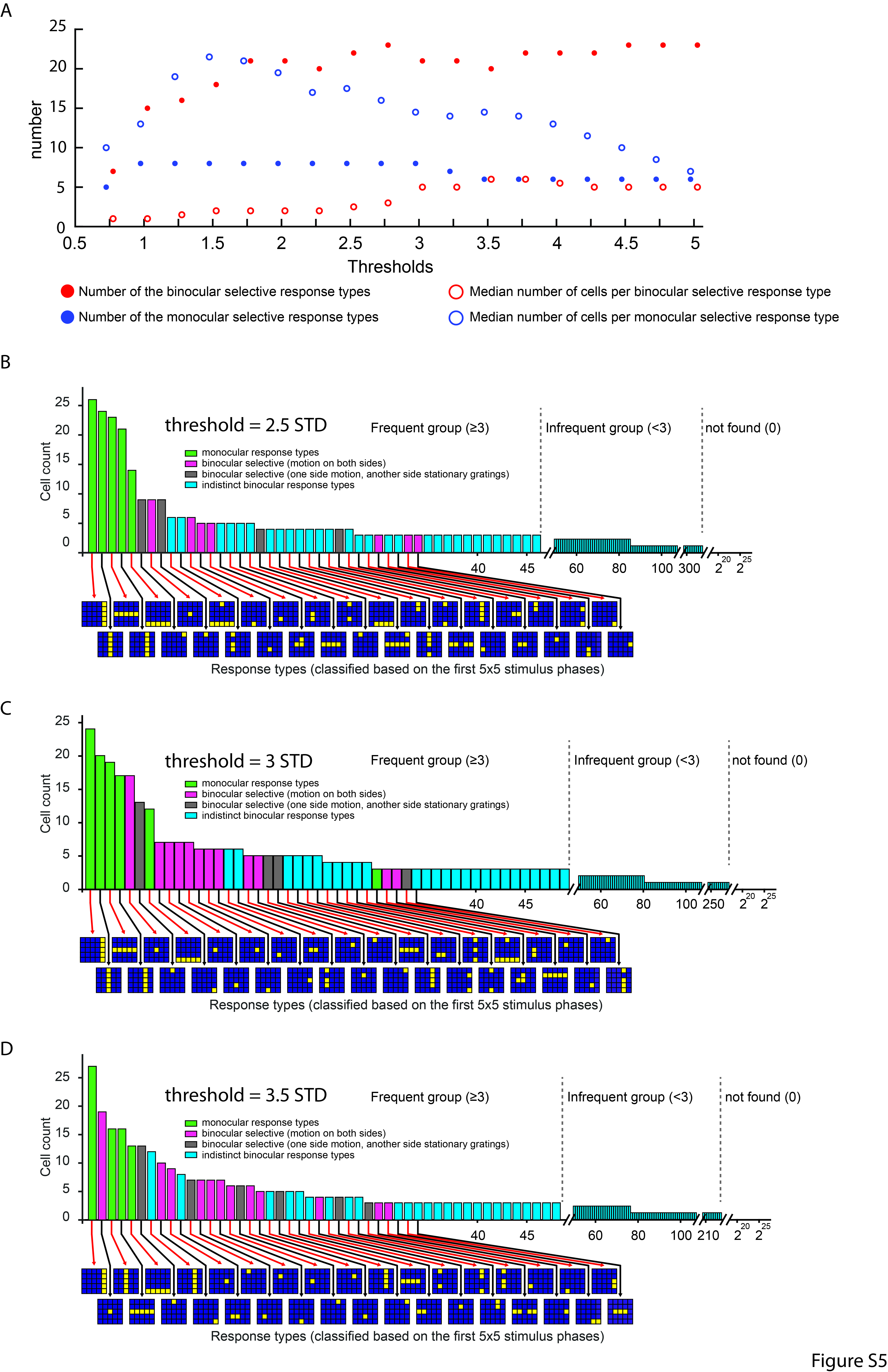

Supplement: Supplementary file 5 — Figure S5. (related to Fig. 2). Binary response type analysis with different thresholds. (A) Response type analysis using 10 different thresholds. While the median number of identified neurons per response type is affected by the choice of threshold, the number of identified binocular-selective and monocular response types is only mildly affected by threshold choice. (B) A lower threshold of 2.5 × STD + mean was used. (C) Analysis for the original threshold of 3 × STD + mean. (D) Analysis using a higher threshold of 3.5 × STD + mean. STD and mean correspond to the standard deviation and mean of the calcium signal ΔF/F during the stationary phases (related to Fig. 2e). (JPG 7371 kb) [file 12915_2019_648_MOESM5_ESM.jpg]

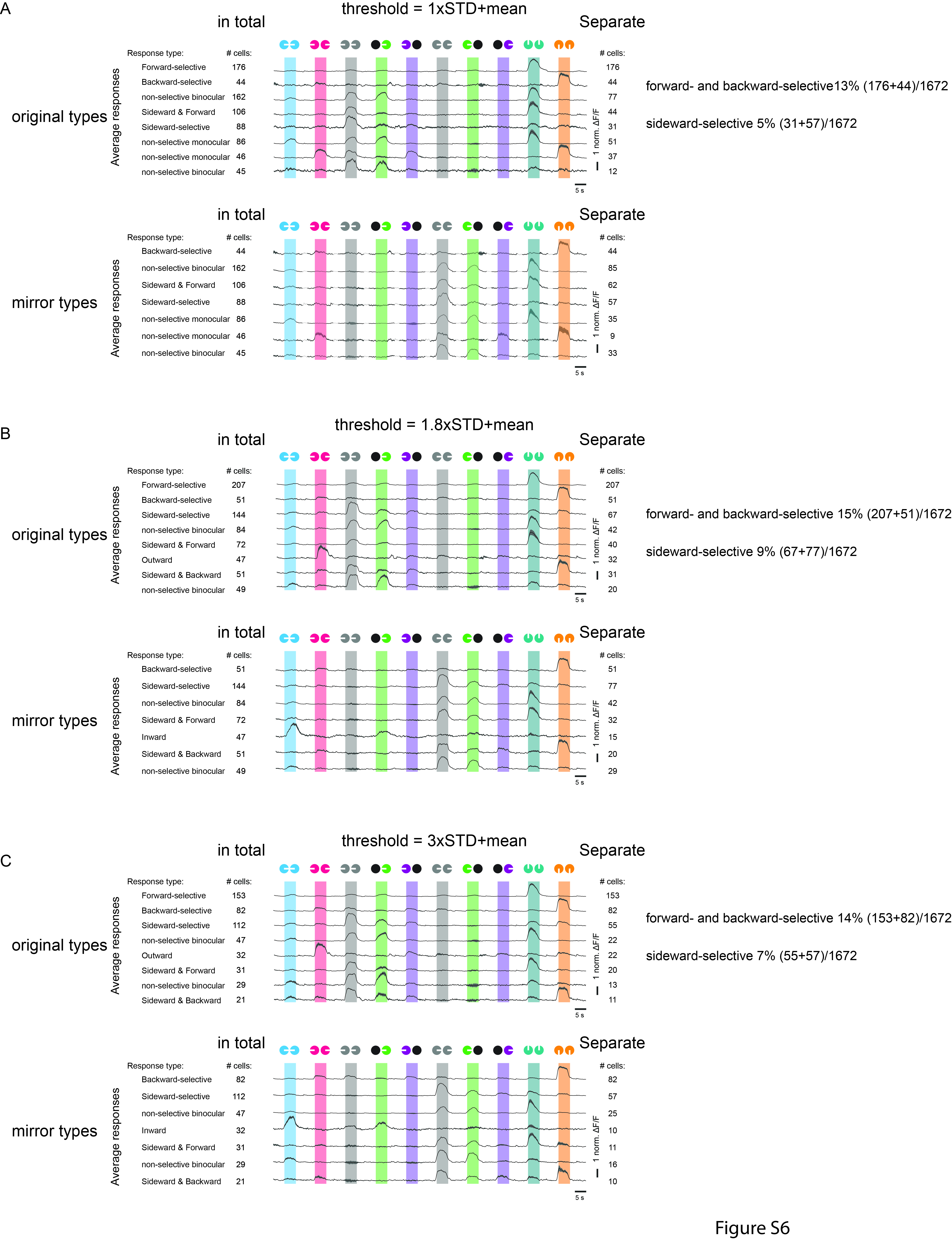

Supplement: Supplementary file 6 — Figure S6. (related to Fig. 3). Effect of changing the activity threshold on identified response profiles of the neurons from the data of Naumann et al. Response profiles of the eight most frequent response types are illustrated in (A, B) using different thresholds. The visual stimulus-evoked calcium signals were detected with thresholds, 1 × STD + mean (A), 1.8 × STD + mean (B) and 3 × STD + mean (C). In this study we used a threshold of 1 × STD, while the previous study used 1.8 × STD. Except for FW and BW response types, in each panel, the indicated neuron numbers on the left side of the plot correspond to the sums of the response type pairs active during leftward and rightward motion. On the right side of the plot, the numbers indicated the neuron numbers of each individual response type (i.e., without mirror-symmetrical response type). The mirror-symmetrical response type pairs were plotted separately on the upper and lower panel. The proportions of the forward-, backward-, and sideward-selective neurons are indicated on the right of each panel. STD, standard deviation of the calcium signal ΔF/F during the stationary phases; mean, mean of the calcium signal ΔF/F during the stationary phases. The icons and colors are identical to those in Fig. 3. (JPG 6249 kb) [file 12915_2019_648_MOESM6_ESM.jpg]

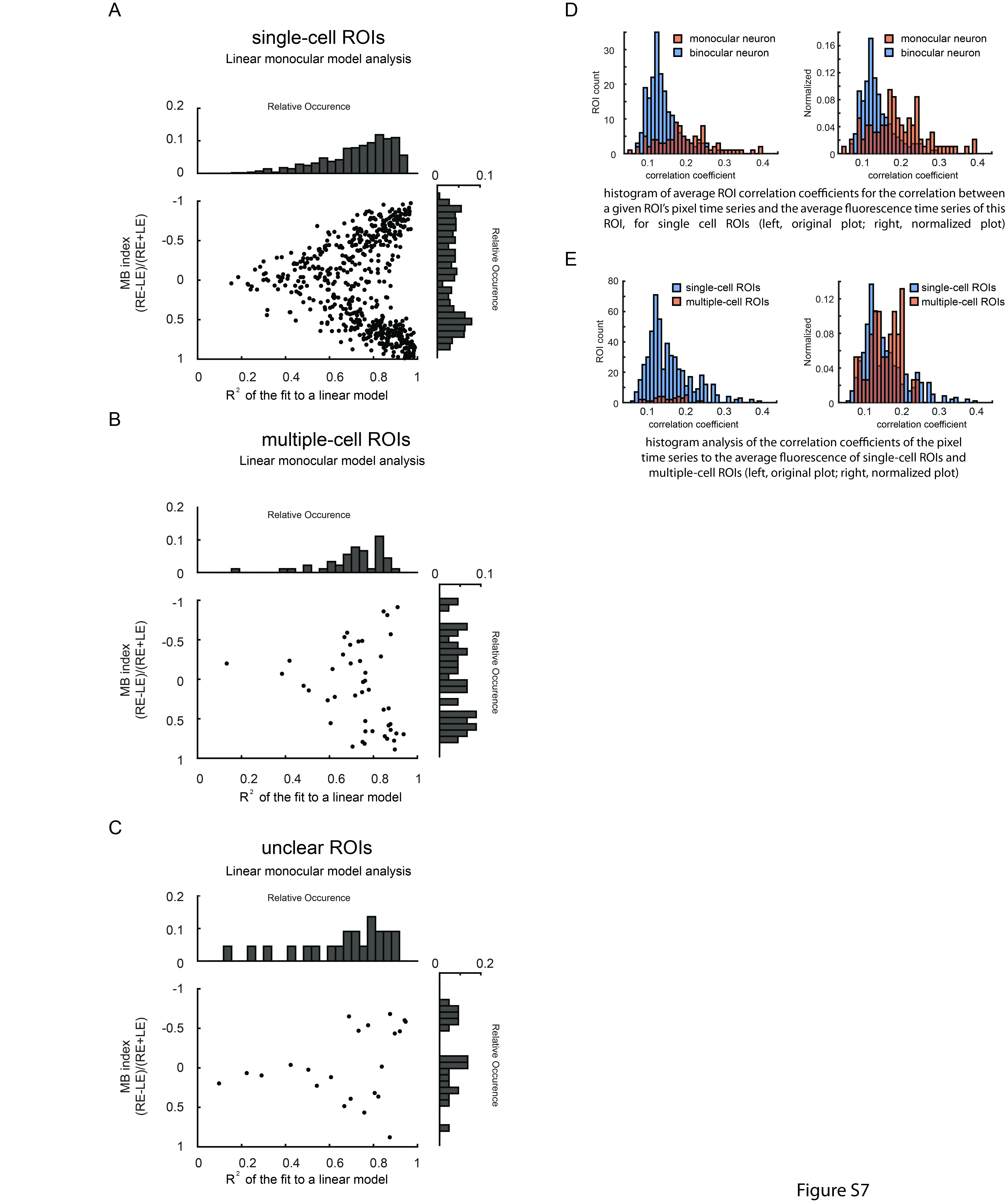

Supplement: Supplementary file 7 — Figure S7. (Related to Fig. 2). Pixel-wise correlation analysis suggests that manually drawn ROIs correspond to single neurons in most cases in the binocular stimulation dataset. (A–C) Linear model analysis of the binocular experiment data for the single-cell ROIs (A), multiple-cell ROIs (B) and unclear ROIs (C) (related to Additional file 1: Figure S1G). (D) Histogram of average ROI correlation coefficients for the correlation between a given ROI’s pixel time series and the average fluorescence time series of this ROI, for single-cell ROIs from the 49 frequent response types (left, original plot; right, normalized plot). Blue, binocular neurons (binocular selective or indistinct neurons); pale red, monocular neurons. (E) histogram analysis of the correlation coefficients of the pixel time series to the average fluorescence of single-cell ROIs and multiple-cell ROIs from the 49 frequent response types (left, original plot; right, normalized plot). (JPG 5536 kb) [file 12915_2019_648_MOESM7_ESM.jpg]

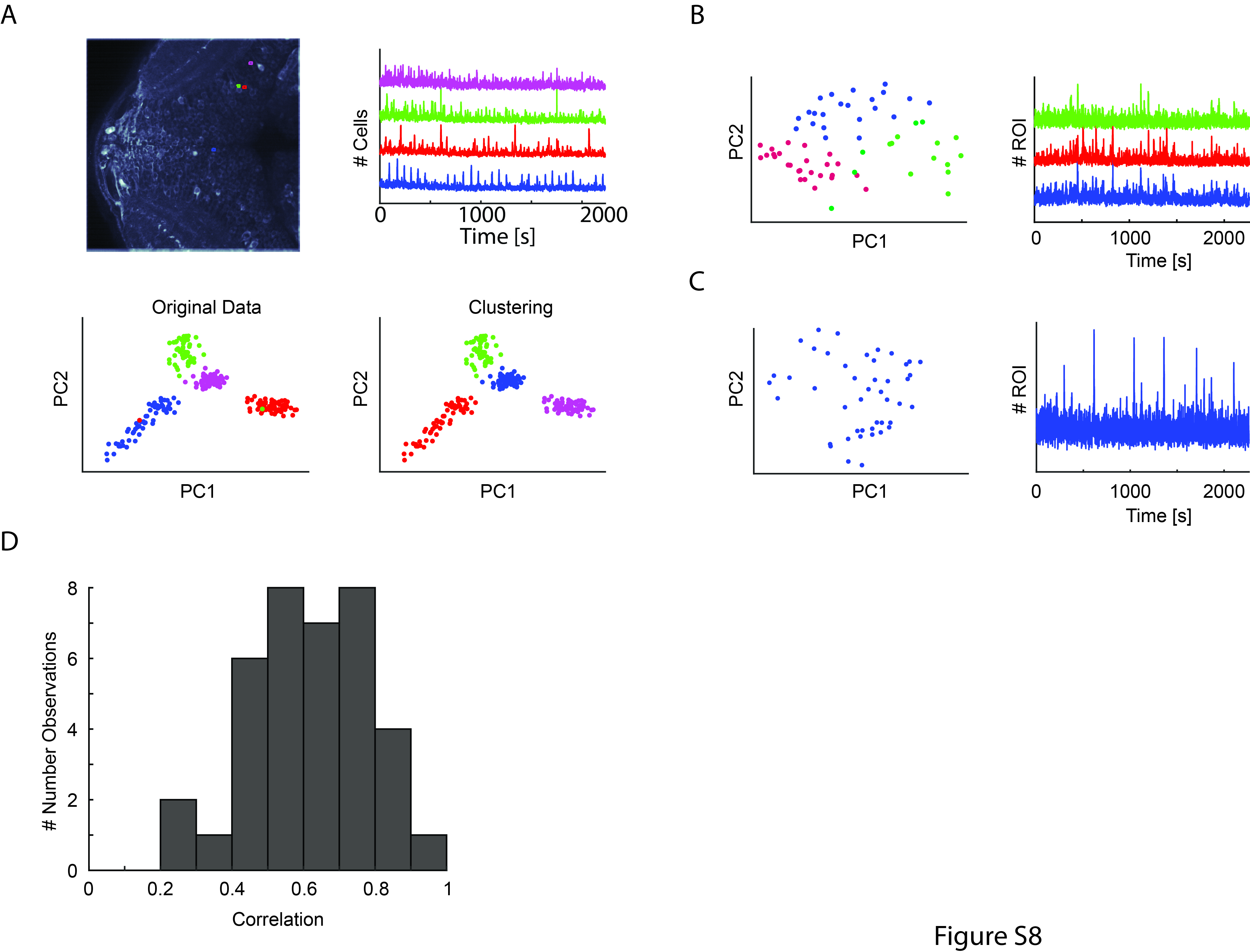

Supplement: Supplementary file 8 — Figure S8. (Related to Fig. 2). Clustering of local pixel correlations reveals highly correlated activity patterns for cells that were classified as multiple ROIs. (A) Examples demonstrating the effectiveness of the method. Four ROIs that were manually identified to be single cells (their activity traces are shown on the right) were combined and each pixel of these ROIs was correlated with each other. Subsequently, we performed principal component analysis (PCA) and expectation maximization clustering, which automatically segmented even spatially close neurons as independent units. Pixels were plotted according to the first two principal components (PCs) at the bottom, illustrating that that the developed algorithm (PCA and clustering) successfully identified the correspondence of the pixels to their original ROIs (lower left: color code based on manual ROI selection, lower right: color code based on assigned cluster identity). (B) Example cluster analysis showing a potential “multiple-cell” ROI (see manual analysis in Additional file 7: Figure S7) that was split into three separate clusters by the algorithm (left); and the accompanying activity traces for each cluster (shown on the right). (C) Example cluster analysis showing an ROI that wasn’t split, and its corresponding activity trace. (D) Quantification of the average correlation of the mean ROI traces resulting from the clustering (e.g., average correlation of the three traces shown in (B)). Correlation is overall high, suggesting that there is no major signal contamination even for neurons that were manually assigned to potentially contain multiple-cell activity. (JPG 2823 kb) [file 12915_2019_648_MOESM8_ESM.jpg]
